# Host cellular parthanatos induced by *Brucella* effector BspA is a critical determinant for its virulence

**DOI:** 10.1016/j.cellin.2026.100376

**Published:** 2026-09-25

**Authors:** Li-Bo Cao, Jin-Ke Yang, Xuehui Wang, Xiao-Wen Wang, Shanquan Wu, Zhen-Zhen Li, Jia-Jing Wang, Dongsheng Lei, Jian Xu, YueFeng Chu, Xin Wu, Yu-Lin Yang

**Affiliations:** a State Key Laboratory for Animal Disease Control and Prevention, College of Veterinary Medicine, Lanzhou University, Lanzhou Veterinary Research Institute, Chinese Academy of Agricultural Sciences, Lanzhou, Gansu, 730000, China; b Gansu Province Research Center for Basic Disciplines of Pathogen Biology, Lanzhou Veterinary Research Institute, Chinese Academy of Agricultural Sciences, Lanzhou, Gansu, 730046, China; c Institute of Respiratory Health and Multimorbidity, West China Hospital, Sichuan University, Chengdu, Sichuan, 610041, China; d Key Laboratory of Magnetism and Magnetic Functional Materials (Lanzhou University), School of Physical Science and Technology, Electron Microscopy Centre of Lanzhou University, Lanzhou University, Lanzhou, Gansu, 730000, China

**Keywords:** *Brucella*, Parthanatos, Pathogenesis, BspA, Olaparib

## Abstract

Brucellosis is a serious zoonotic disease caused by *Brucella* infection, which poses a significant threat to global public health and the livestock industry. The pathogenic mechanisms of *Brucella* remain poorly understood, which limits the development of effective prevention and treatment strategies. Here we report that *Brucella* induces copper (Cu)-dependent host parthanatos through its secreted effector BspA. BspA is localized to the ER and mitochondria and promotes ER–mitochondria contacts. Upon binding to Cu, BspA forms large irregular aggregates that disrupt ER and mitochondrial membrane structures, resulting in Ca^2+^ overload and excessive production of reactive oxygen species (ROS) in the mitochondria, ultimately triggering host cellular parthanatos. BspA-deficiency or pharmacological inhibition of parthanatos suppresses *Brucella*-induced cell death, and reduces *Brucella* loads and inflammatory response in infected mice. Our findings suggest that BspA-induced parthanatos plays a critical role in pathogenicity of *Brucella* and provides potential molecular targets and candidate therapeutics for the treatment of brucellosis.

## Highlights

• BspA is an effector required for *Brucella*-induced parthanatos.
• BspA binds to copper (Cu) to form large irregular aggregates, which disrupt mitochondrial/ER structures.
• Disruption of mitochondrial/ER structures induces excessive ROS to trigger parthanatos.
• Deficiency of BspA or pharmacological inhibition of parthanatos suppresses pathogenesis of *Brucella*.

## Graphical abstract

**Figure:**
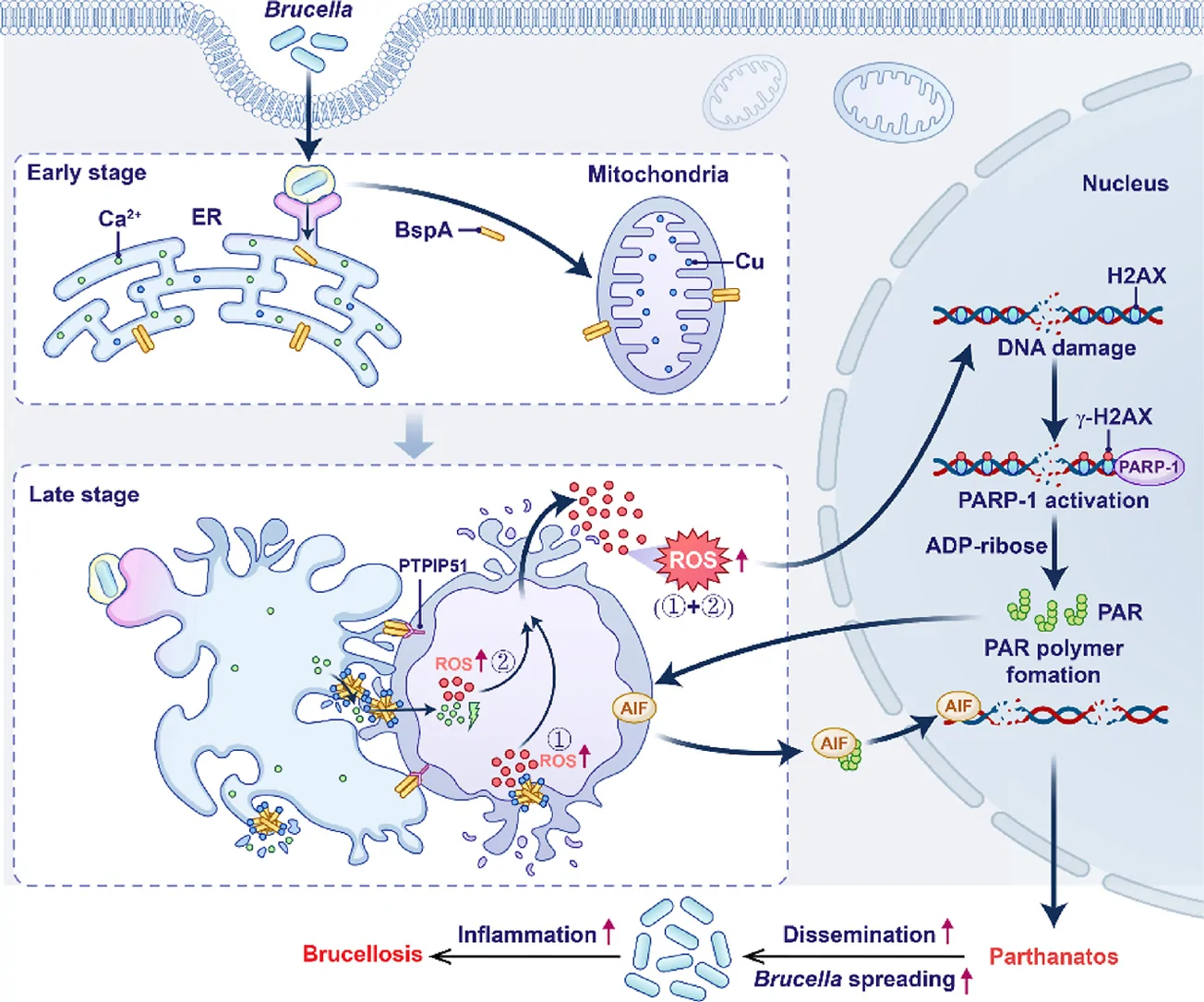

## 1 Introduction

Brucellosis is a widespread zoonotic disease caused by intracellular bacteria of the genus *Brucella* (Atluri et al., 2011). Currently, brucellosis remains endemic in more than 170 countries and regions, causing over 2 million new human infections annually and an estimated economic loss of approximately 3 billion USD to the livestock industry (Dadar et al., 2021; Laine et al., 2023; McDermott et al., 2013). This poses a serious threat to public health and the sustainable development of animal husbandry. Consequently, brucellosis has been designated as a priority zoonosis of concern by both the World Health Organization (WHO) and the World Organization for Animal Health (WOAH). As a classical intracellular pathogen, *Brucella* has been widely reported to induce host cell death during infection (Barquero-Calvo et al., 2015; Gutiérrez-Jiménez et al., 2019; Yang, Wang, et al., 2024; Zhang et al., 2024). However, the precise pathways and molecular mechanisms underlying *Brucella*-induced cell death remain enigmatic, and the development of vaccines and specific therapeutic drugs still lacks effective molecular targets. Therefore, elucidating cell death pathways triggered by *Brucella* and identifying key bacterial effectors or host factors involved will not only provide new insights into mechanisms of *Brucella* pathogenesis but also offer potential strategies for development of safe vaccines and specific therapeutic interventions against brucellosis.

Parthanatos is a distinct form of programmed cell death first described in 2008 (Andrabi et al., 2008). Excessive generation of reactive oxygen species (ROS) or nitric oxide (NO) induces oxidative or nitrosative stress, causing extensive DNA strand breaks, which trigger rapid phosphorylation of the histone H2A variant H2AX (callled γ-H2AX) on chromatin near the damage sites (Rogakou et al., 1998). DNA damage also overactivates poly (ADP-ribose) polymerase-1 (PARP-1), which in turn catalyzes the synthesis of large amounts of poly(ADP-ribose) (PAR) polymers in the nucleus (de Murcia & Ménissier de Murcia, 1994; Virág, 2005; Wang et al., 2016; Zhang et al., 1994). PAR translocates from the nucleus to mitochondria, where it binds to apoptosis-inducing factor (AIF), leading to the release of AIF from mitochondria and its translocation to the nucleus. Nuclear AIF then mediates large-scale DNA fragmentation and chromatin condensation, ultimately resulting in cell death called parthanatos (Martínez-Morcillo et al., 2021; Murata et al., 2019; Wang et al., 2011, 2016). Unlike apoptosis, parthanatos is caspase-independent and primarily driven by ROS/NO-mediated DNA strand breaks and PARP-1 hyperactivation (Hadian & Stockwell, 2023). The hallmark features of parthanatos include formation of γ-H2AX, which reflects extensive DNA double-strand breaks, and abnormal accumulation of PAR, which reflects PARP-1 hyperactivation (Collins et al., 2020; Wang et al., 2009, 2016).

Parthanatos has been primarily reported in the context of neurodegenerative diseases, where oligomerization of distinct molecules triggers neuronal parthanatos and is considered a key contributor to disease progression (Cheignon et al., 2018; Kam et al., 2018; Yang, Guttman, et al., 2024). However, whether parthanatos also plays an important role during *Brucella* infection that causes severe host disease has not yet been reported. In this study, we identified the *Brucella* encoded secreted protein BspA as a critical effector to induce host cellular parthanatos. BspA is localized to both the endoplasmic reticulum (ER) and mitochondrial membranes, predominantly exists as small aggregates with molecular weight sizes larger than 400 kDa. Upon binding to copper (Cu), BspA forms large irregular aggregates that disrupt the integrity of ER and mitochondrial membrane. The damaged ER releases large amounts of calcium ions (Ca^2+^), which enters mitochondria via BspA-PTPIP51-mediated ER–mitochondria contacts, resulting in mitochondrial Ca^2+^ overload and production of ROS. Additionally, induction of mitochondrial membrane damage by BspA also contributes to ROS production. Production of excessive ROS eventually leads to host cellular parthanatos. Importantly, deficiency of BspA or pharmacological inhibition of parthanatos markedly reduces *Brucella*-induced splenomegaly and inflammation, thereby alleviating disease symptoms in mice. Our findings suggest that *Brucella* BspA-triggered host cellular parthanatos is a critical mechanism of brucellosis, and targeting BspA-triggered parthanatos can be effective for treatment of brucellosis.

## 2 Results

### 2.1 *Brucella* induces copper-dependent parthanatos of host cells

Numerous studies have reported that the pathogenicity of bacterial pathogens is closely associated with host cell death (Lu et al., 2025; Qiang et al., 2023; Sun et al., 2015). To investigate the roles of *Brucella*-induced host cell death in pathogenicity, the mouse macrophage cell line RAW264.7 was infected with the highly virulent *B. abortus* S2308, followed by analysis of lactate dehydrogenase (LDH) release, one of the indicators of cell death (Cummings & Schnellmann, 2004). The results showed that cell death in RAW264.7 cells increased significantly with the prolonged duration of *Brucella* infection (Fig. 1(A)). To identify the major cell death pathways induced by *Brucella*, we examined the effects of inhibitors targeting cuproptosis (tetrathiomolybdate, TTM), ferroptosis (ferrostatin-1, Fer-1), apoptosis (Q-VD-OPH, QV), necroptosis (necrostatin-1, Nec-1), and parthanatos (olaparib) on *Brucella*-induced host cell death. The results showed that TTM and olaparib markedly inhibited *Brucella*-induced host cell death, suggesting that *Brucella* might trigger cuproptosis or parthanatos of host cells (Fig. 1(B)). The occurrence of cuproptosis is usually associated with mitochondrial Cu overload (Cong et al., 2025; Tsvetkov et al., 2022). However, *Brucella* infection did not affect the level of intracellular and mitochondrial Cu in host cells (Fig. 1(C)), suggesting that the observed cell death might not be typical cuproptosis. Instead, *Brucella* infection induced hallmark events of parthanatos, characterized by abnormal accumulation of PAR and phosphorylation of H2AX (γ-H2AX), both of which were markedly suppressed by TTM and olaparib (Fig. 1(D)). In these experiments, neither TTM nor olaparib significantly affected the level of the *Brucella* outer membrane protein 28 (Omp28) or the intracellular bacterial burden, indicating that their effects are unlikely to be attributable to inhibition of intracellular *Brucella* replication (Fig. 1(D) and (E)).

**Fig. 1:**
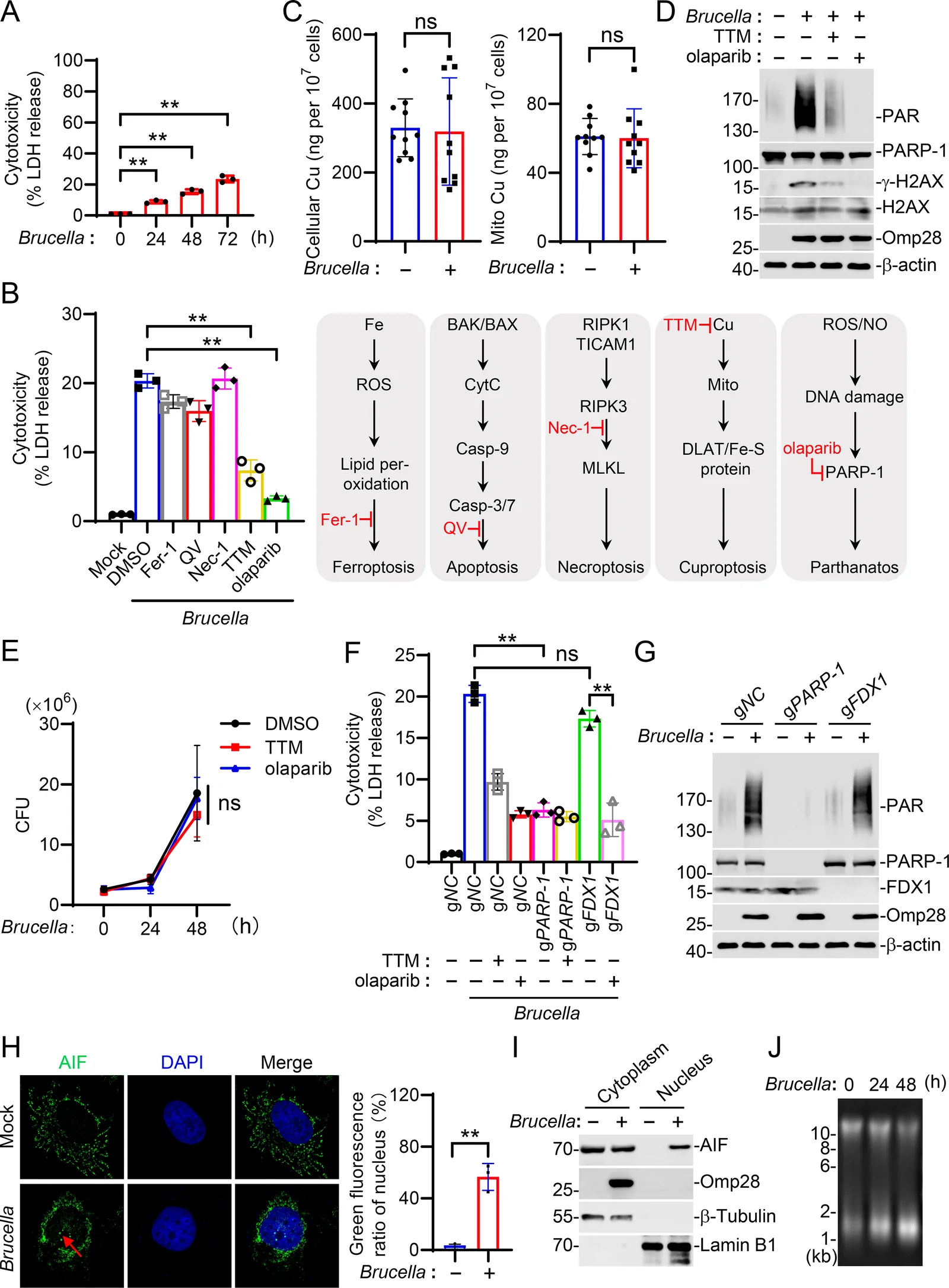
***Brucella*****induces copper-dependent parthanatos.** (A) *Brucella* induces host cell death. RAW264.7 cells (2 × 10^5^) were infected with *B. abortus* S2308 (MOI = 200) for the indicated times, followed by lactate dehydrogenase (LDH) release analysis. (B) Effects of cell death pathway inhibitors on *Brucella*-induced host cell death. RAW264.7 cells (2 × 10^5^) were infected with *B. abortus* S2308 (MOI = 200) for 48 h in the absence or presence of cell death pathway inhibitors tetrathiomolybdate (TTM, 40 μM), ferrostatin-1 (Fer-1, 20 μM), Q-VD-OPH (QV, 10 μM), necrostatin-1 (Nec-1, 20 μM), or olaparib (20 μM), followed by LDH release analysis. (C) Effects of *Brucella* infection on cellular or mitochondrial copper (Cu) levels in host cells. Mitochondria were isolated from RAW264.7 cells (4 × 10^6^) after 48 h of *B. abortus* S2308 (MOI = 200) infection, and cellular or mitochondrial Cu levels were measured by ICP-MS. Data are representative of 10 samples per group (*n* = 10). (D) Effects of TTM or olaparib on *Brucella*-induced parthanatos. RAW264.7 cells (2 × 10^5^) were left uninfected or infected with *B. abortus* S2308 (MOI = 200) for 48 h in the absence or presence of TTM (40 μM) or olaparib (20 μM), followed by immunoblotting analysis with the indicated antibodies. (E) Effects of TTM or olaparib on intracellular replication of *Brucella*. RAW264.7 cells (2 × 10^5^) were infected with *B. abortus* S2308 for 4 h at MOI = 200. At indicated time points, cells were lysed with 0.5% Triton X-100 and CFUs were enumerated. (F) Effects of PARP-1- or FDX1-deficiency on *Brucella*-induced host cell death. Non-targeting control (g*NC*), PARP-1-deficient (g*PARP-1*), or FDX1-deficient (g*FDX1*) RAW264.7 cells (2 × 10^5^) were left uninfected or infected with *B. abortus* S2308 (MOI = 200) for 48 h in the absence or presence of TTM (40 μM) or olaparib (20 μM), followed by LDH release analysis. (G) Effects of PARP-1- or FDX1-deficiency on *Brucella*-induced parthanatos. PARP-1- or FDX1-deficient RAW264.7 cells (2 × 10^5^) were left uninfected or infected with *B. abortus* S2308 (MOI = 200) for 48 h, followed by immunoblotting analysis with the indicated antibodies. (H) Effects of *Brucella* infection on AIF localization. HeLa cells (2 × 10^4^) were left uninfected or infected with *B. abortus* S2308 (MOI = 200) for 36 h and then fixed for immunostaining before subjected to confocal microscopy. Representative immunofluorescence images are shown on the left, with the green fluorescence signal representing AIF. The right panel shows the percentage of cells exhibiting nuclear AIF fluorescence signals, quantified among 20 randomly selected cells per field. (I) Effects of *Brucella* infection on AIF nuclear translocation. HeLa cells (2 × 10^5^) were left uninfected or infected with *B. abortus* S2308 (MOI = 200) for 36 h, followed by nuclear and cytoplasmic fractionation. The resulting fractions were analyzed by immunoblotting with the indicated antibodies. (J) Effects of *Brucella* infection on DNA fragmentation. Genomic DNA was extracted from HeLa cells (2 × 10^5^) 24 or 48 h after *B. abortus* S2308 (MOI = 200) infection using a genomic DNA extraction kit, and DNA integrity was assessed by agarose gel electrophoresis. Data shown in (A), (B), (E), (F) and (H) are mean ± SD (*n* = 3) from three independent experiments. All the experiments were repeated at least twice with similar results. ns, nonsignificant, ∗∗*P* < 0.01.

FDX1 and PARP-1 are key regulators of cuproptosis and parthanatos respectively, and their deficiencies markedly suppress the occurrence of the corresponding cell death (Kam et al., 2018; Tsvetkov et al., 2022). To further determine whether *Brucella* primarily induces cuproptosis or parthanatos of host cells, we investigated the effects of deficiency of either FDX1 or PARP-1 on *Brucella*-induced death of RAW264.7 cells. The results indicated that deficiency of PARP-1 (g*PARP-1*) but not FDX1 (g*FDX1*) markedly inhibited *Brucella*-induced death of RAW264.7 cells. Moreover, olaparib retained the ability to inhibit *Brucella*-induced death in FDX1-deficient cells (Fig. 1(F)). Consistently, deficiency of PARP-1 but not FDX1 markedly inhibited *Brucella*-induced accumulation of PAR (Fig. 1(G)). In addition to PARP-1 activation, parthanatos is also characterized by AIF translocation to the nucleus and DNA fragmentation (Fatokun et al., 2014). Infection experiments demonstrated that *Brucella* infection induced AIF translocation from the mitochondria to the nucleus (Fig. 1(H) and (I)), accompanied by pronounced DNA fragmentation (Fig. 1(J)). Collectively, these results suggest that *Brucella* infection primarily induces parthanatos but not cuproptosis of host cells. Since the Cu chelator TTM inhibited *Brucella*-induced parthanatos and previous studies suggest that TTM can affect various cellular processes by reducing the levels of intracellular free Cu (Tsang et al., 2021), it is possible that accumulation of intracellular free Cu is required for *Brucella*-induced parthanatos.

### 2.2 BspA is an effector required for *Brucella*-induced parthanatos

To identify the effector proteins required for *Brucella*-induced parthanatos of host cells, we constructed mammalian expression plasmids for 31 *Brucella*-secreted proteins that were reported in previous studies (Louche et al., 2023; Luizet et al., 2021; Myeni et al., 2013). We transfected these plasmids individually into HEK293T cells and assessed their abilities to induce cell death by the CCK-8 assay. The results showed that BspA, encoded by *BAB1_0678* gene, induced the most severe cell death compared to other *Brucella*-secreted proteins, and this cell death was effectively inhibited by both TTM and olaparib (Fig. S1A). Further experiments demonstrated that BspA induced cell death in a dose-dependent manner in HEK293T cells (Fig. S1B). Consistent with *Brucella*-induced cell death, TTM and olaparib, but not inhibitors of other types of cell death, inhibited BspA-induced cell death (Fig. 2(A)). Deficiency of PARP-1 but not FDX1 markedly inhibited BspA-induced cell death, and olaparib retained the ability to inhibit BspA-induced death of FDX1-deficient cells (Fig. 2(B)). Deficiency of PARP-1 but not FDX1 also markedly inhibited BspA-induced accumulation of PAR in HEK293T cells (Fig. 2(C)). Moreover, ectopic expression of BspA promoted AIF nuclear translocation and induced DNA fragmentation (Fig. 2(D–G)). These results suggest that BspA induces cellular parthanatos. To further determine whether BspA-induced parthanatos is dependent on accumulation of intracellular Cu, we established a cellular model with low intracellular free Cu level by ectopically expressing the Cu-exporting protein ATP7B in HEK293T cells. In these cells, treatment of Cu^2+^ failed to induce cell death (Fig. 2(H)), and cell death induced by ectopic expression of BspA was markedly reduced compared to control cells (Fig. 2(I)). These results suggest that BspA induces Cu-dependent parthanatos of host cells.

**Fig. 2:**
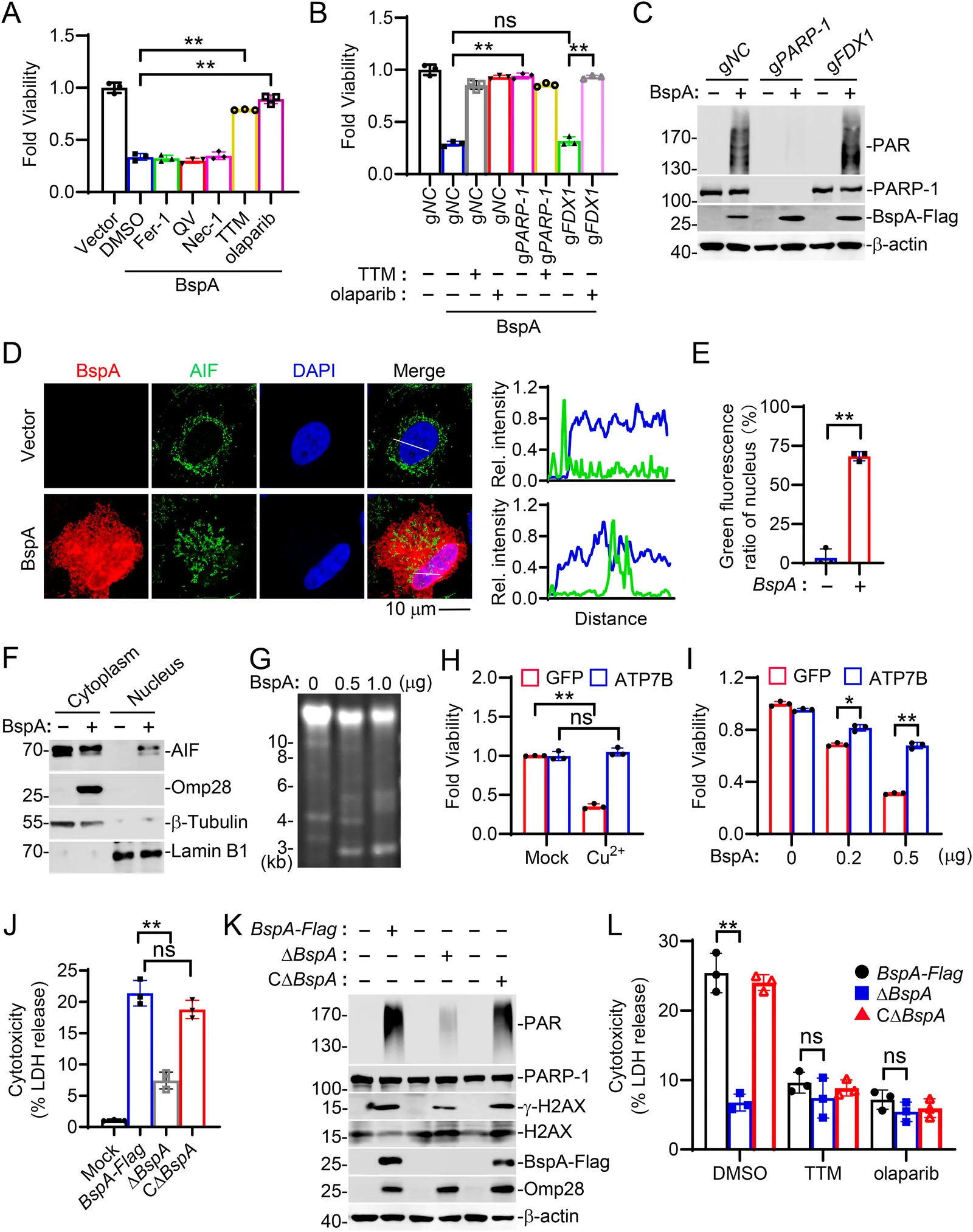
**BspA is an effector required for*****Brucella*****-induced parthanatos.** (A) Effects of cell death pathway inhibitors on BspA-induced host cellular death. HEK293T cells (2 × 10^5^) were transfected with an empty vector or BspA plasmid (1 μg) for 48 h in the absence or presence of cell death pathway inhibitors ferrostatin-1 (Fer-1, 20 μM), Q-VD-OPH (QV, 10 μM), necrostatin-1 (Nec-1, 20 μM), tetrathiomolybdate (TTM, 40 μM) and olaparib (20 μM), followed by CCK-8 analysis. (B) Effects of PARP-1- or FDX1-deficiency on BspA-induced host cell death. PARP-1- or FDX1-deficient HEK293T cells (2 × 10^5^) were transfected with an empty vector or BspA plasmid (1 μg) for 48 h in the absence or presence of TTM (40 μM) or olaparib (20 μM), followed by CCK-8 analysis. (C) Effects of PARP-1- or FDX1-deficiency on BspA-induced parthanatos of host cells. PARP-1- or FDX1-deficient HEK293T cells (2 × 10^5^) were transfected with an empty vector or BspA plasmid (1 μg) for 48 h, followed by immunoblotting analysis with the indicated antibodies. (D-E) Effects of BspA on AIF localization. HeLa cells (2 × 10^4^) were transfected with the indicated plasmids for 36 h and then fixed for immunostaining before subjected to confocal microscopy (D). The percentage of cells exhibiting nuclear AIF fluorescence signals was quantified among 20 randomly selected cells per field (E). (F) Effects of BspA on AIF nuclear translocation. HeLa cells (2 × 10^5^) were transfected with an empty vector or BspA plasmid (1 μg) for 36 h, followed by nuclear and cytoplasmic fractionation. The resulting fractions were analyzed by immunoblotting with the indicated antibodies. (G) Effects of BspA on DNA fragmentation. Genomic DNA was extracted from HEK293T cells (2 × 10^5^) 24 h after transfection with BspA plasmid (0, 0.5 and 1.0 μg) using a genomic DNA extraction kit, and DNA integrity was assessed by agarose gel electrophoresis. (H) Effects of ATP7B on Cu^2+^-induced cell death. HEK293T cells (2 × 10^5^) were transfected with GFP or ATP7B plasmid (1 μg) for 24 h, followed by Cu^2+^ (10 μM) treatment for 8 h, and cell viability was measured using CCK-8 assay. (I) Effects of ATP7B on BspA-induced cell death. HEK293T cells (2 × 10^5^) were transfected with the indicated plasmids for 48 h, followed by CCK-8 assay. (J) Effects of BspA-deficiency on *Brucella*-induced cell death. RAW264.7 cells (2 × 10^5^) were left uninfected or infected with *BspA-Flag*, Δ*BspA* or CΔ*BspA* strains (MOI = 200) for 48 h, followed by LDH release analysis. (K) Effects of BspA-deficiency on *Brucella*-induced parthanatos of host cells. RAW264.7 cells (2 × 10^5^) were left uninfected or infected with *BspA-Flag*, Δ*BspA* or CΔ*BspA* strains (MOI = 200) for 48 h, followed by immunoblotting analysis with the indicated antibodies. (L) Effects of TTM or olaparib on *Brucella*-induced host cell death. RAW264.7 cells (2 × 10^5^) were left uninfected or infected with *BspA-Flag*, Δ*BspA* or CΔ*BspA* strains (MOI = 200) for 48 h in the absence or presence of TTM (40 μM) or olaparib (20 μM), followed by LDH release analysis. Data shown in (A), (B), (E), (H-J) and (L) are mean ± SD (*n* = 3) from three independent experiments. All the experiments were repeated at least twice with similar results. ns, nonsignificant, ∗*P* < 0.05, ∗∗*P* < 0.01.

To investigate whether BspA is required for *Brucella*-induced parthanatos, we generated *B. abortus* S2308 with Flag-tagged *BspA* (*BspA-Flag*), *BspA*-deficiency (Δ*BspA*), and Δ*BspA* strain complemented with Flag-tagged *BspA* (CΔ*BspA*) through homologous recombination or plasmid complementation (Fig. S2A). The expression of BspA-Flag was confirmed by immunoblotting analysis in cells infected with the three engineered *Brucella* strains (Fig. S2B). All three strains exhibited growth rates comparable to the wild-type *B. abortus* S2308 *in vitro* (Fig. S2C). In the subsequent experiments, we used the *BspA-Flag* strain instead of the wild-type *Brucella* strain to facilitate detection of BspA. We next compared the abilities of these three engineered *Brucella* strains to induce death of host cells. The results showed that the Δ*BspA* strain had markedly reduced ability to induce death of RAW264.7 cells, whereas the CΔ*BspA* strain markedly induced death of host cells to a level comparable to that induced by the *BspA-Flag* strain (Fig. 2(J)). Consistently, immunoblotting analysis showed that deficiency of BspA impaired *Brucella*-induced accumulation of PAR and γ-H2AX without significantly affecting the expression level of the *Brucella* outer membrane protein Omp28, whereas the CΔ*BspA* strain restored accumulation of PAR and γ-H2AX to levels comparable to those induced by the *BspA-Flag* strain. (Fig. 2(K)). To further determine whether TTM and olaparib specifically inhibit BspA-mediated cell death, we examined their effects on cell death induced by the Δ*BspA* strain. Our results showed that both TTM and olaparib effectively inhibited cell death induced by the *BspA-Flag* and the CΔ*BspA* strains, but not that induced by the Δ*BspA* strain (Fig. 2(L)). These results suggest that BspA is a key effector responsible for *Brucella*-induced parthanatos of host cells.

### 2.3 BspA binds to copper to form large irregular aggregates

Our earlier results demonstrated that *Brucella* induced parthanatos of host cells through its effector protein BspA in a Cu-dependent manner (Fig. 2(H–I)). To investigate the underlying mechanisms, we first examined whether BspA is capable of binding Cu. The cell lysates expressing BspA-Flag protein were incubated with Cu^2+^-charged or Cu^2+^-free resins. The proteins bound to Cu^2+^ were eluted from the resins through competitive binding of Cu^2+^ by imidazole, and the eluted fractions were subsequently analyzed by immunoblotting. The results showed that BspA efficiently bound to Cu^2+^, comparable to the classical Cu^2+^-binding protein lipoylated DLAT (Tsvetkov et al., 2022) (Fig. 3(A)). To further investigate potential biochemical changes of BspA upon Cu binding, recombinant BspA was expressed in a prokaryotic system and purified to near homogeneity (Fig. 3(B)). When subjected to size-exclusion chromatography on Superdex 200 Increase 10/300 GL column, which separates proteins in the 10-300 kDa range, BspA predominantly eluted in the void volume fractions that did not penetrate the packed resins, indicating that it may form high-molecular-weight assemblies beyond the resolving capacity of the resins (Fig. 3(C)). Notably, purified BspA rapidly formed precipitates upon incubation with Cu^2+^. The supernatant obtained after centrifugation was subjected to size-exclusion chromatography, revealing that the high-molecular-weight BspA assemblies were largely removed by precipitation and were not present in soluble forms (Fig. 3(D)). Consistently, native PAGE followed by immunoblotting analysis indicated that the purified BspA formed soluble high-molecular-weight assemblies exceeding 400 kDa. Incubation of these assemblies with Cu^2+^, but not Ca^2+^, caused transition of most of the proteins to precipitates (Fig. 3(E)). To directly visualize the soluble and Cu^2+^-induced insoluble high molecular weight complexes of BspA, transmission electron microscopy was performed. The results showed that soluble BspA assemblies formed aggregates with diameters of approximately 30 nm. Upon incubation with Cu^2+^, these soluble aggregates were disrupted, ultimately resulting in the formation of large irregular aggregates (Fig. 3(F)). These results suggest that purified BspA exists as soluble aggregates *in vitro*, and Cu^2+^ binding disrupts these structures and results in formation of insoluble large irregular aggregates.

**Fig. 3:**
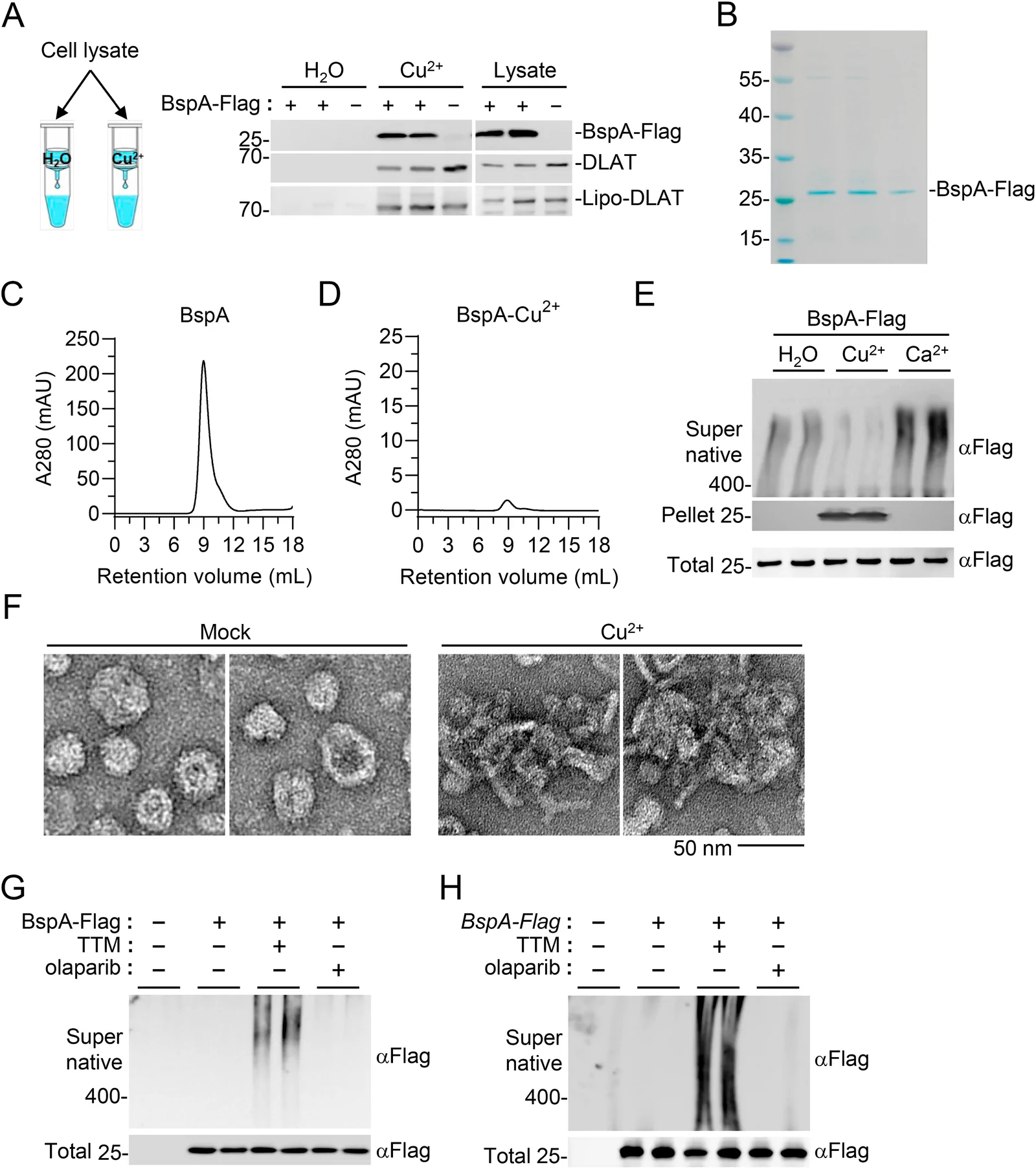
**BspA binds to copper to form large irregular aggregates.** (A) Binding of BspA to Cu^2+^. HEK293T cells (2 × 10^5^) were transfected with an empty vector or BspA plasmid (1 μg) for 24 h. The cell lysates were incubated with either Cu^2+^-loaded or Cu^2+^-free resins. The proteins eluted from the respective resins were analyzed by immunoblotting with the indicated antibodies. (B) Purification of BspA protein. BspA protein was purified using a prokaryotic expression system, and its purity was assessed by SDS-PAGE, followed by Ccoomassie brilliant blue staining. (C-D) Analysis of purified BspA protein by size-exclusion chromatography. Purified BspA protein was left untreated (C) or incubated with Cu^2+^ (10 μM) (D) prior to size-exclusion chromatography to assess its purity and aggregates. (E) Effects of Cu^2+^ on the solubility of BspA aggregates. Purified BspA protein was left untreated or incubated with Cu^2+^ (10 μM) or Ca^2+^ (10 μM) for 30 min followed by centrifugation at 12 000×*g* for 10 min at 4 °C. The supernatants were analyzed by native PAGE to assess BspA aggregates, while the pellets were analyzed by SDS-PAGE. (F) Analysis of purified BspA protein by transmission electron microscopy. Purified BspA protein was left untreated or incubated with Cu^2+^ (10 μM) before observation by transmission electron microscopy. (G) Effects of TTM or olaparib on a fraction of BspA with molecular weights exceeding 400 kDa in a mammalian overexpression system. HEK293T cells (5 × 10^5^) were transfected with an empty vector or BspA plasmid (1 μg) for 48 h in the absence or presence of TTM (40 μM) or olaparib (20 μM). The cell lysates were centrifuged at 12 000×*g* for 10 min at 4 °C. The supernatants were analyzed by native PAGE to assess BspA aggregates, while the totals were analyzed by SDS-PAGE to detect expression of BspA. (H) Effects of TTM or olaparib on a fraction of BspA with molecular weights exceeding 400 kDa in *Brucella*-infected cells. RAW264.7 cells (2 × 10^5^) were left uninfected or infected with *BspA-Flag* strain (MOI = 200) for 48 h in the absence or presence of TTM (40 μM) or olaparib (20 μM). The cell lysates were centrifuged at 12 000×*g* for 10 min at 4 °C. The supernatants were analyzed by native PAGE to assess BspA aggregates, while the totals were analyzed by SDS-PAGE to detect expression of BspA. All the experiments were repeated at least twice with similar results.

To determine whether BspA similarly forms Cu-dependent insoluble large aggregates in cells, we examined the properties of BspA in BspA-expressed HEK293T cells and RAW264.7 cells infected with the *BspA-Flag* strain. Immunoblotting analysis indicated that BspA with a molecular weight exceeding 400 kDa was barely detectable in the soluble cellular fractions, whether it was ectopically expressed in HEK293T cells or endogenously expressed in BspA-Flag-infected RAW264.7 cells. Treatment of the cells with the Cu chelator TTM, rather than olaparib, caused transition of a fraction of BspA with molecular weights exceeding 400 kDa to soluble cellular fractions (Fig. 3(G) and (H)). Since Cu is abundant in cell culture medium and within the cells, our results suggest that BspA exists in part as insoluble large aggregates in the cells.

### 2.4 BspA disrupts the structures of mitochondria and ER

To investigate which cellular organelle structures might be affected by BspA aggregation in cells, we first examined the subcellular localization of BspA using confocal microscopy. The results showed that BspA was co-localized extensively with the ER and partially with the mitochondria at 48 h after transfection (Fig. 4(A)), but exhibited little co-localization with the Golgi (Fig. 4(B)) at 24 h after transfection. Notably, ectopic expression of BspA caused marked morphological alterations in subcellular structures at 48 h post-transfection, including swelling of the ER and mitochondria (Fig. 4(A)). Consistently, immunoelectron microscopy confirmed the localization of BspA to the ER and mitochondria at 18 h post-transfection (Fig. 4(C)). In our study, we confirmed its ER localization and further found that BspA also localized to mitochondria. Furthermore, transmission electron microscopy showed that ectopic expression of BspA caused severe disruption of ER and mitochondrial structures at 48 h post-transfection (Fig. 4(D)). Although previous studies reported that BspA predominantly localized to ER at 24 h post-transfection without causing obvious alterations in ER morphology (Kambarev et al., 2023; Myeni et al., 2013), our study revealed that ectopic expression of BspA for 48 h resulted in pronounced ER structural remodeling. These findings suggest that BspA-induced organelle remodeling may occur in a time-dependent manner. At the early stage, BspA primarily exhibits ER localization. However, BspA may progressively disrupt ER homeostasis and membrane dynamics with sustained expression and accumulation, ultimately leading to ER structural perturbation. Moreover, we noted that ER and mitochondria appeared to form more extensive contacts at 24 h after BspA transfection. Quantitative analysis by transmission electron microscopy showed an increase in the number of ER–mitochondria contacts (Fig. 4(D)). However, ectopic expression of BspA failed to induce the swelling and structural disruption of the ER and mitochondria in the presence of TTM, indicating that BspA-triggered swelling and disruption of the ER and mitochondria is dependent on intracellular free Cu (Fig. 4(E)). In addition, we observed that BspA formed numerous small punctate aggregates in the presence of TTM (Fig. 4(E)). This observation is consistent with our *in vitro* electron microscopy findings, which showed that Cu^2+^ promoted the formation of insoluble BspA aggregates, whereas BspA predominantly existed as soluble aggregates in the absence of Cu^2+^ (Fig. 3(F)).

**Fig. 4:**
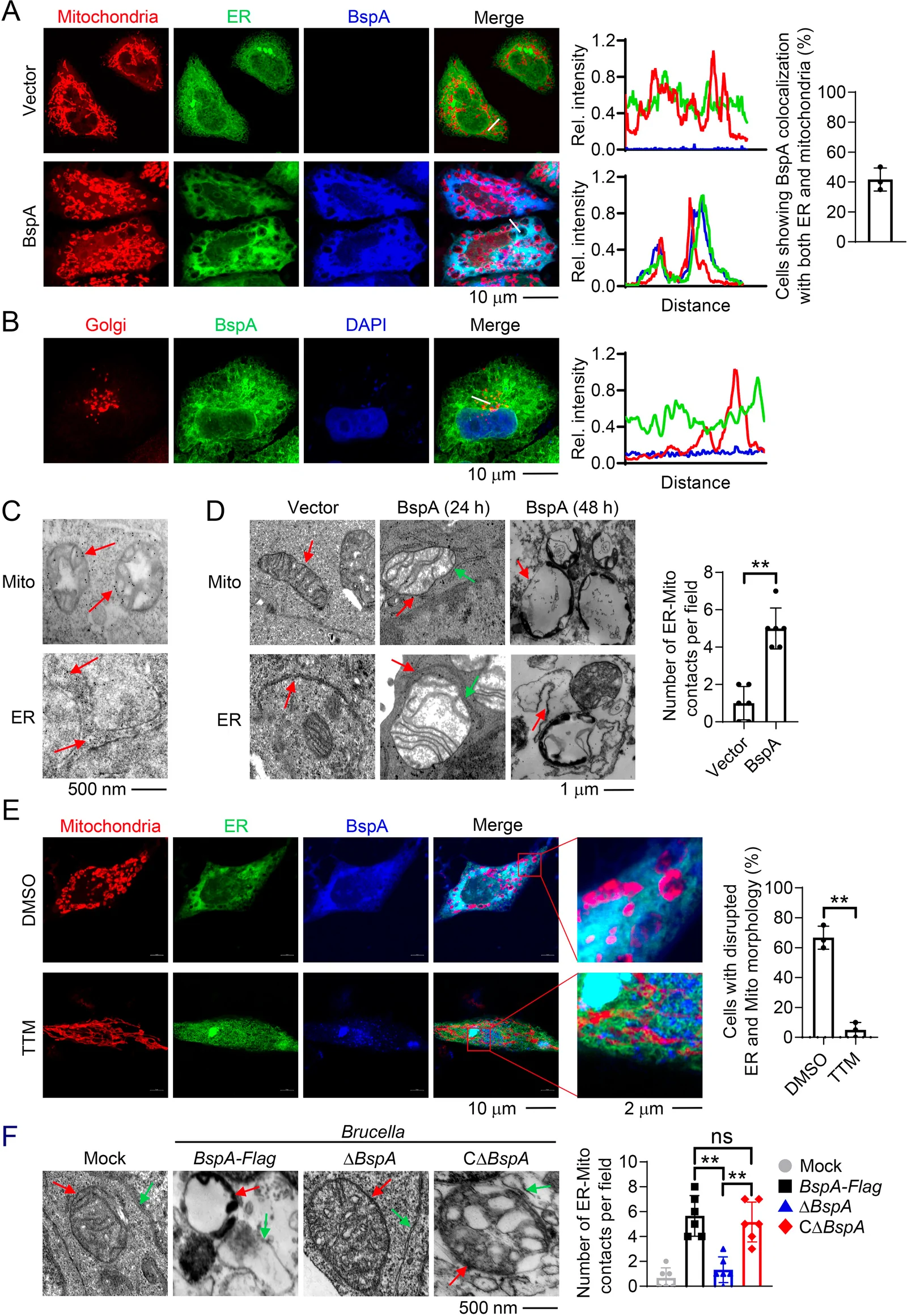
**BspA disrupts the structures of mitochondria and ER.** (A-B) Confocal microscopy analysis of BspA subcellular localization. HeLa cells (2 × 10^4^) were transfected with the indicated plasmids for 24 or 48 h and then fixed for immunostaining before subjected to confocal microscopy. The percentage of cells exhibiting BspA colocalization with both ER and mitochondria quantified among 20 randomly selected cells per field (A), but exhibited little co-localization with the Golgi (B). (C) Immunoelectron microscopy analysis of BspA subcellular localization. HeLa cells (4 × 10^6^) were transfected with the BspA plasmid (2 μg) for 18 h and then fixed for immunostaining before subjected to immunoelectron microscopy. (D) Effects of BspA on mitochondrial and ER structures. HeLa cells (4 × 10^6^) were transfected with an empty vector or BspA plasmid (2 μg) for the indicated times and then fixed before observation by transmission electron microscopy. The red arrows indicate ER and mitochondria, while the green arrows indicate ER–mitochondria contacts. Quantification of ER–mitochondria contacts in each independent field of view (15 × 15 μm) is shown at right. Each dot represents one field of view. Data are representative of 6 independent fields of view per group (*n* = 6). (E) Effects of TTM on BspA-induced swelling of the ER and mitochondria. HeLa cells (2 × 10^4^) were transfected with the indicated plasmids for 48 h in the absence or presence of TTM (40 μM) and then fixed for immunostaining before subjected to confocal microscopy. The percentage of cells with disrupted ER and mitochondria morphology was quantified among 20 randomly selected cells per field. (F) Effects of BspA-deficiency on structures of ER and mitochondria in *Brucella*-infected cells. HeLa cells (4 × 10^6^) were left uninfected or infected with *BspA-Flag*, Δ*BspA* or CΔ*BspA* strains (MOI = 200) for 48 h, followed by transmission electron microscopy analysis. The red arrows indicate mitochondria, while the green arrows indicate ER. Quantification of ER–mitochondria contacts in each independent field of view (15 × 15 μm) is shown at right. Each dot represents one field of view. Data are representative of 6 independent fields of view per group (*n* = 6). Data shown in (A) and (E) are mean ± SD (*n* = 3) from three independent experiments. All the experiments were repeated at least twice with similar results. ns, nonsignificant, ∗*P* < 0.05, ∗∗*P* < 0.01.

To further investigate the effects of endogenous BspA on host cellular organelle structures, subcellular structures of cells infected with the *BspA-Flag*, Δ*BspA*, or CΔ*BspA* strain were analyzed by transmission electron microscopy. Consistent with above observations, infection with either the *BspA-Flag* or CΔ*BspA* strain induced marked swelling of both the ER and mitochondria, whereas infection with the Δ*BspA* strain caused no marked morphological alterations in host cells (Fig. 4(F)). Collectively, these results suggest that BspA is localized to the ER and mitochondria in *Brucella*-infected host cells, where it forms large insoluble aggregates upon binding to Cu, which disrupt ER and mitochondrial membrane structures and induces their swelling.

### 2.5 ER**–**mitochondria contacts mediated by BspA-PTPIP51 promotes cellular parthanatos

In our earlier confocal and transmission electron microscopy observation, we showed that the disrupted ER and mitochondria appeared to form extensive contacts (Fig. 4(A) and (D)). Given that ER–mitochondria contacts have been reported to play a role in regulating cell death (Area-Gomez et al., 2012; Filadi et al., 2016; Namba, 2019), we further investigated the molecular mechanisms underlying BspA-induced ER–mitochondria contacts. Previous studies have reported that ER–mitochondria contacts are primarily mediated through four classical molecular interaction modes: (1) IP3R3-VDAC, (2) MFN2-MFN1, (3) VAPB-PTPIP51, and (4) BAP31-TOMM40 (Larrañaga-SanMiguel et al., 2025; Wilson & Metzakopian, 2021). We used small interfering RNAs (siRNAs) to knock down the ER-localized components involved in these four contact modes and then evaluated their effects on BspA-induced cell death. The results indicated that knockdown of IP3R3, MFN2, VAPB and BAP31 had no marked effects on cell viability or BspA-induced cell death, indicating that BspA-triggered ER–mitochondria contacts are not dependent on the four classical ER–mitochondria contact modes or not required for BspA-induced cell death (Fig. 5(A) and Fig. S3A). Given that BspA is predominantly localized to the ER, we hypothesized that it might interact with mitochondrial outer-membrane proteins to promote ER–mitochondria contacts. To test this hypothesis, we examined the interaction between BspA and the mitochondrial partners of the four classical contact pairs. The results indicated that BspA interacted with PTPIP51 but not VDAC1, MFN1 or TOMM40 in a mammalian overexpression system (Fig. 5(B)). Endogenous co-immunoprecipitation experiments indicated that BspA was associated with endogenous PTPIP51 in *BspA-Flag*-infected RAW264.7 cells (Fig. 5(C)). Confocal microscopy indicated that BspA colocalized with PTPIP51 (Fig. 5(D)). Since our earlier results indicated that BspA was partially localized to mitochondria (Fig. 4(A) and (C)), and PTPIP51 is localized to mitochondria (De Vos et al., 2012), these observations raised the question of whether the mitochondrial localization of BspA is dependent on PTPIP51. Therefore, we investigated whether BspA can be localized to mitochondria in PTPIP51-deficient cells. The results indicated that depletion of PTPIP51 by siRNA had no marked effects on the localization of BspA in mitochondria (Fig. 5(E–F) and Fig. S3B). However, further experiments showed that knockdown of PTPIP51 suppressed *Brucella*-induced ER–mitochondria contacts (Fig. 5(G)). These results suggest that the interaction between BspA and PTPIP51 promotes ER–mitochondria contacts.

**Fig. 5:**
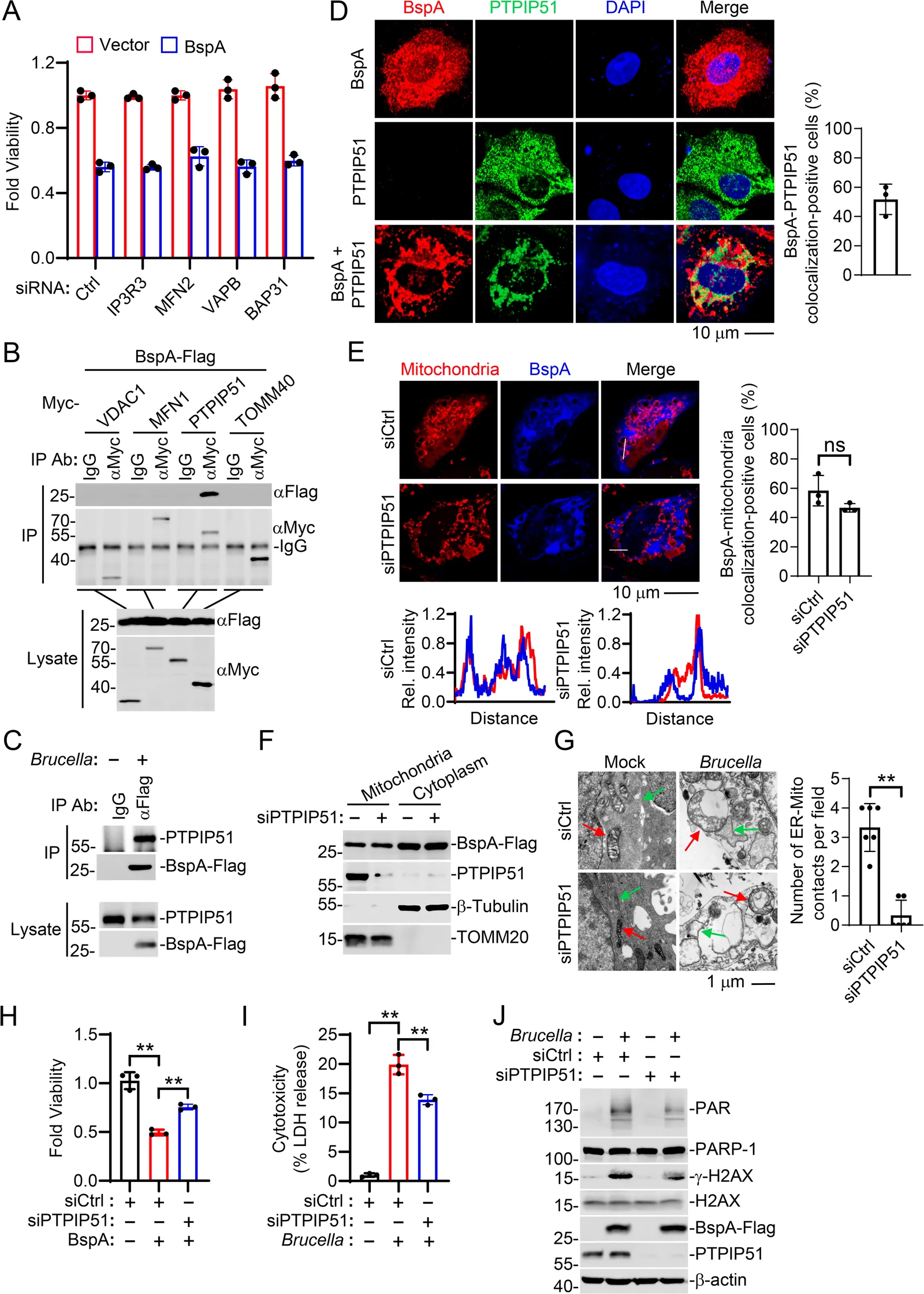
**ER–mitochondria contacts mediated by BspA-PTPIP51 promotes parthanatos.** (A) Effects of knocking down four classical ER-localized contact proteins on BspA-induced cell death. HEK293T cells (1 × 10^5^) were first transfected with the indicated small interfering RNAs (siRNAs) (50 nM) for 24 h, then transfected with an empty vector or BspA plasmid (1 μg) for an additional 48 h before CCK-8 assay. (B) The interaction of BspA with four classical mitochondria-localized contact proteins. HEK293T cells (5 × 10^6^) were transfected with the indicated plasmids for 24 h and then lysed for co-immunoprecipitation with control IgG or anti-Myc antibody, followed by immunoblotting analysis with the indicated antibodies. (C) The endogenous interaction of BspA with PTPIP51. RAW264.7 cells (2 × 10^7^) were left uninfected or infected with *BspA-Flag* strain (MOI = 200) for 48 h and then lysed for co-immunoprecipitation with control IgG or anti-Flag antibody, followed by immunoblotting analysis with the indicated antibodies. (D) Co-localization of BspA with PTPIP51. HeLa cells (2 × 10^4^) were transfected with the indicated plasmids for 24 h and then fixed for immunostaining before subjected to confocal microscopy. The percentage of cells with BspA-PTPIP51 colocalization was quantified among 20 randomly selected cells per field. (E) Effects of knocking down PTPIP51 on the mitochondrial localization of BspA. HeLa cells (2 × 10^4^) were transfected with siRNA of negative control (Ctrl) or PTPIP51 (50 nM) for 24 h, followed by transfection with the BspA plasmid (1 μg) for an additional 48 h. The cells were then fixed for immunostaining before subjected to confocal microscopy. The percentage of cells with BspA-mitochondria colocalization was quantified among 20 randomly selected cells per field. (F) Effects of knocking down PTPIP51 on the mitochondrial localization of BspA. HeLa cells (2 × 10^5^) were transfected with BspA plasmid (1 μg) for 36 h, followed by mitochondria and cytoplasmic fractionation. The resulting fractions were analyzed by immunoblotting with the indicated antibodies. (G) Effects of knocking down PTPIP51 on *Brucella*-induced ER–mitochondria contacts. HeLa cells (6 × 10^5^) were transfected with siRNA of Ctrl or PTPIP51 (50 nM) for 24 h, followed by infection with *BspA-Flag* strain for an additional 48 h. The cells were then fixed before observation by transmission electron microscopy. The red arrows indicate mitochondria, while the green arrows indicate ER. Quantification of ER–mitochondria contacts in each independent field of view (15 × 15 μm) is shown at right. Each dot represents one field of view. Data are representative of 6 independent fields of view per group (*n* = 6). ∗∗*P* < 0.01. (H) Effects of knocking down PTPIP51 on BspA-induced host cell death. HeLa cells (1 × 10^5^) were transfected with siRNA of Ctrl or PTPIP51 (50 nM) for 24 h and then transfected with an empty vector or BspA plasmid (1 μg) for an additional 48 h before CCK-8 assay. (I) Effects of knocking down PTPIP51 on *Brucella*-induced host cell death. HeLa cells (1 × 10^5^) were transfected with siRNA of Ctrl or PTPIP51 (50 nM) for 24 h and then left uninfected or infected with *BspA-Flag* strain (MOI = 200) for an additional 48 h, followed by LDH release analysis. (J) Effects of knocking down PTPIP51 on *Brucella*-induced parthanatos of host cells. HeLa cells (1 × 10^5^) were transfected with siRNA of Ctrl or PTPIP51 (50 nM) for 24 h and then infected with *BspA-Flag* strain (MOI = 200) for an additional 48 h, followed by immunoblotting analysis with the indicated antibodies. Data shown in (A), (D), (E) and (G-I) are mean ± SD (*n* = 3) from three independent experiments. All the experiments were repeated at least twice with similar results. ns, nonsignificant, ∗∗*P* < 0.01.

To further investigate whether BspA-PTPIP51-mediated ER–mitochondria contacts contribute to *Brucella*-induced cell death, we compared the susceptibility of wild-type and PTPIP51-knockdown cells to ectopic expression of BspA or *Brucella* infection. The results indicated that knockdown of PTPIP51 partially reduced BspA- or *Brucella*-induced host cell death (Fig. 5(H) and (I)). Consistently, biochemical analysis indicated that knockdown of PTPIP51 suppressed but did not completely block *Brucella*-induced parthanatos (Fig. 5(J)). Collectively, these results suggest that BspA interacts with PTPIP51 to induce ER–mitochondria contacts, thereby promoting *Brucella*-induced parthanatos of host cells during infection.

### 2.6 BspA induces parthanatos by disrupting homeostasis of mitochondria and ER

Excessive production of ROS or NO is one of the major triggers of parthanatos (Cheignon et al., 2018; Zhang et al., 1994). To explore the mechanism by which BspA induces parthanatos, we examined the effects of BspA on ROS and NO levels in host cells. The results showed that ectopic expression of BspA markedly increased both cellular and mitochondrial ROS production (Fig. 6(A)), whereas it had little effect on NO levels (Fig. 6(B)). Excessive mitochondrial ROS production is typically associated with mitochondrial dysfunction, including loss of membrane potential and impaired ATP synthesis (Ahmed Selim & Wojtovich, 2025; Shadel & Horvath, 2015). As expected, ectopic expression of BspA led to a significant reduction in mitochondrial membrane potential (Δψm) (Fig. 6(C)). The *BspA-Flag* and CΔ*BspA* strains exhibited comparable abilities to suppress ATP synthesis in host cells, whereas the Δ*BspA* strain had markedly reduced ability in suppression of ATP synthesis (Fig. 6(D)). These results suggest that BspA disrupts the homeostasis and physiological functions of mitochondria.

**Fig. 6:**
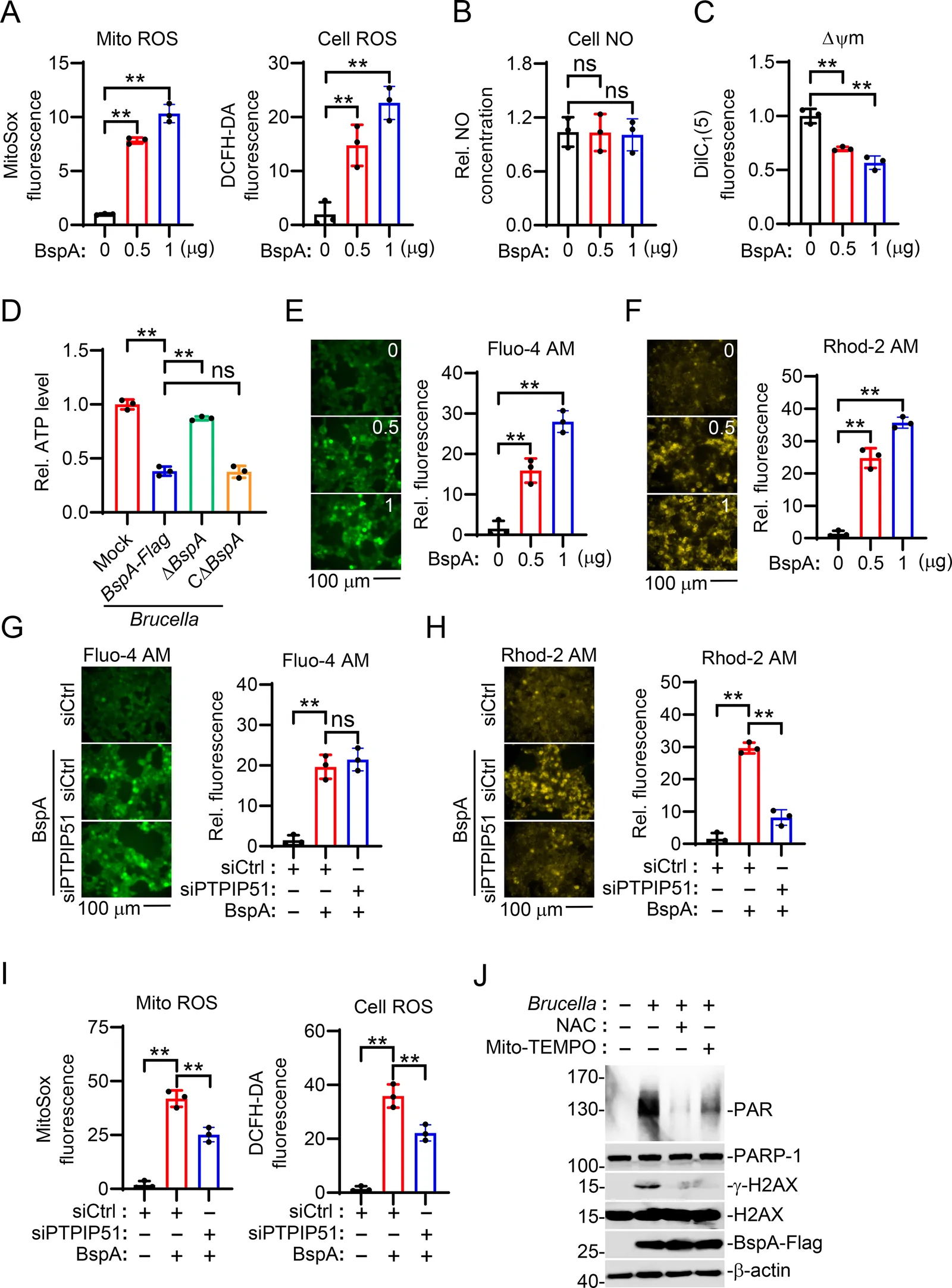
**BspA induces parthanatos by disrupting the homeostasis of mitochondria and ER.** (A) Effects of BspA on cellular and mitochondrial reactive oxygen species (ROS). HEK293T cells (2 × 10^5^) were transfected with BspA plasmids (0, 0.5 and 1.0 μg) for 24 h, and then stained with DCFH-DA (for cell ROS) and MitoSOX (for mitochondrial ROS). Fluorescence intensity was detected to assess ROS levels in cytosol or mitochondria. Quantification of ROS levels in cytosol or mitochondria per field is shown (each dot represents one field). (B) Effects of BspA on intracellular nitric oxide (NO) levels. HEK293T cells (2 × 10^5^) were transfected with BspA plasmids (0, 0.5 and 1.0 μg) for 24 h, followed by measurement of intracellular NO levels. (C) Effects of BspA on the mitochondrial membrane potential of host cells. HEK293T cells (2 × 10^5^) were transfected with BspA plasmids (0, 0.5 and 1.0 μg) for 24 h, followed by measurement of mitochondrial membrane potential (Δψm). (D) Effects of BspA-deficiency on ATP production in host cells. RAW264.7 cells (2 × 10^6^) were left uninfected or infected with *BspA-Flag*, Δ*BspA* or CΔ*BspA* strains (MOI = 200) for 48 h and then lysed for measurement of intracellular ATP levels. (E-F) Effects of BspA on the levels of Ca^2+^ in the cytosol and mitochondria. HEK293T cells (2 × 10^5^) were transfected with BspA plasmids (0, 0.5 and 1.0 μg) for 24 h, and then stained with Fluo-4 AM (for cytosolic Ca^2+^ level) (E) and Rhod-2 AM (for mitochondrial Ca^2+^ level) (F). Fluorescence intensity was detected to assess Ca^2+^ levels in cytosol or mitochondria. Quantification of Ca^2+^ levels in cytosol or mitochondria per field is shown at right (each dot represents one field). (G-H) Effects of knocking down PTPIP51 on BspA-induced increases of Ca^2+^ levels in the cytosol and mitochondria. HEK293T cells (2 × 10^5^) were transfected with siRNA of Ctrl or PTPIP51 (50 nM) for 24 h followed by transfection with an empty vector or BspA plasmid (1 μg) for an additional 48 h. The cells were then stained with Fluo-4 AM (G) and Rhod-2 AM (H). Fluorescence intensity was detected to assess Ca^2+^ levels in cytosol or mitochondria. Quantification of Ca^2+^ levels in cytosol or mitochondria per field is shown at right (each dot represents one field). (I) Effects of knocking down PTPIP51 on the increases of ROS in the cell and mitochondria induced by BspA. HEK293T cells (2 × 10^5^) were transfected with siRNA of Ctrl or PTPIP51 (50 nM) for 24 h followed by transfection with an empty vector or BspA plasmid (1 μg) for an additional 48 h. The cells were then stained with DCFH-DA (right) and MitoSOX (left). Fluorescence intensity was detected to assess ROS production. Quantification of ROS levels in cytosol or mitochondria per field is shown (each dot represents one field). (J) Effects of ROS scavengers on *Brucella*-induced parthanatos of host cells. RAW264.7 cells (2 × 10^7^) were left uninfected or infected with *BspA-Flag* strain (MOI = 200) for 48 h in the absence or presence of N-Acetylcysteine (NAC, 20 μM) or Mito-TEMPO (10 μM) and then lysed for immunoblotting analysis with the indicated antibodies. Data shown in (A-I) are mean ± SD (*n* = 3) from three independent experiments. All the experiments were repeated at least twice with similar results. ns, nonsignificant, ∗∗*P* < 0.01.

In our earlier experiments, we observed that BspA disrupted ER membrane integrity and induced ER swelling (Fig. 4(A) and (D)). Since ER serves as the main intracellular Ca^2+^ reservoir, its structural disruption results in extensive Ca^2+^ leakage into the cytosol, which subsequently causes Ca^2+^ overload of mitochondria and triggers ROS production (Bagur & Hajnóczky, 2017; Du et al., 2024; Yu et al., 2025). To investigate whether BspA-induced ER damage led to mitochondrial Ca^2+^ overload and thereby promoting ROS generation, we first measured cytosolic and mitochondrial Ca^2+^ levels in BspA-expressing cells. The results showed that ectopic expression of BspA increased Ca^2+^ levels in the cytosol (Fig. 6(E)) as well as in the mitochondria (Fig. 6(F)). However, knockdown of PTPIP51 did not affect BspA-induced cytosolic Ca^2+^ accumulation (Fig. 6(G)), but reduced approximately three-quarters of mitochondrial Ca^2+^ elevation (Fig. 6(H)). These results suggest that BspA-PTPIP51-mediated ER–mitochondria contacts may create a high local concentration of Ca^2+^ near mitochondria due to Ca^2+^ leakage from damaged ER, thereby facilitating mitochondrial Ca^2+^ uptake and resulting in Ca^2+^ overload of mitochondria.

To determine the link between mitochondrial Ca^2+^ overload and ROS production, we examined the effects of PTPIP51 knockdown on BspA-induced ROS production. The results showed that knockdown of PTPIP51 suppressed approximately one-third of BspA-induced ROS production in both the cytosol and mitochondria (Fig. 6(I)). These results indicated that BspA-PTPIP51-mediated ER–mitochondria contacts elevated mitochondrial Ca^2+^ levels, which in turn promoted mitochondrial ROS production. Further experimental results showed that treatment with the ROS scavenger N-Acetylcysteine (NAC) or the mitochondria-targeted antioxidant Mito-TEMPO effectively reduced *Brucella*-induced parthanatos (Fig. 6(J)), indicating that ROS generated from BspA-mediated disruption of ER and mitochondrial homeostasis are key factors triggering parthanatos. Numerous studies have reported that mitochondrial Ca^2+^ overload is one of the key factors triggering apoptosis (Giorgi et al., 2012; Orrenius et al., 2015). However, our experimental results showed that mitochondrial Ca^2+^ overload induced by BspA did not trigger apoptosis of host cells (Fig. S4A and B). This is consistent with our observation that the apoptosis inhibitor QV did not affect BspA-induced cell death (Fig. 2(A)). Collectively, these results suggest that BspA triggers parthanatos via two routes: 1) BspA is localized in mitochondria and directly disrupts mitochondrial membrane structures upon binding to Cu; 2) BspA is localized to ER, promotes ER–mitochondria contacts via its interaction with PTPIP51. This facilitates Ca^2+^ uptake by mitochondria upon its release from ER disrupted by BspA, leading to increased synthesis of ROS by mitochondria.

### 2.7 BspA-deficiency of *Brucella* inhibits pathogenesis

To investigate the roles of BspA-induced parthanatos in pathogenicity of *Brucella*, BALB/c mice were intraperitoneally infected with *BspA-Flag*, Δ*BspA* or CΔ*BspA* strain. At 21 days post-infection, spleens from the infected mice were evaluated for parthanatos. Consistent with the findings *in vitro*, deficiency of BspA attenuated *Brucella*-induced accumulation of PAR and formation of γ-H2AX in the spleen (Fig. 7(A)). Immunohistochemistry experiments also showed that deficiency of BspA attenuated *Brucella*-induced PAR formation in the spleen (Fig. 7(B)). These results suggest that BspA mediates parthanatos in Brucella-infected mice.

**Fig. 7:**
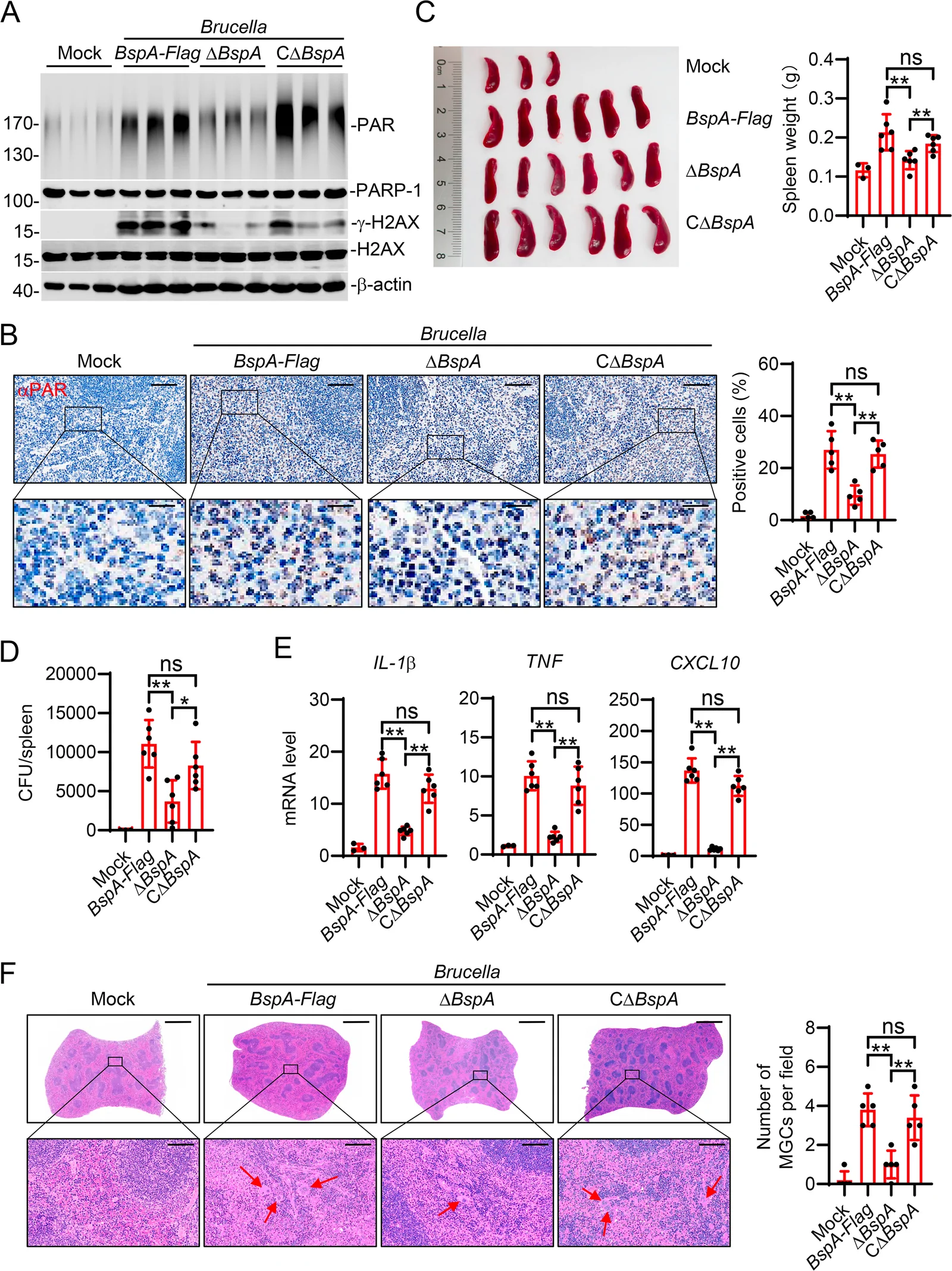
**BspA-deficiency of*****Brucella*****inhibits pathogenesis.** (A) Effects of BspA-deficiency on *Brucella*-induced cellular parthanatos in spleen. Spleen tissues from mice infected with *BspA-Flag*, Δ*BspA* or CΔ*BspA* strains (3 × 10^5^ CFU) were harvested 21 days post-infection and subjected to immunoblotting analysis with the indicated antibodies. (B) Immunohistochemical analysis of spleens. Spleen tissues from mice infected with *BspA-Flag*, Δ*BspA* or CΔ*BspA* strains (3 × 10^5^ CFU) were harvested 21 days post-infection. Tissue sections were stained with anti-PAR antibody for immunohistochemical examination. Quantification of positive cells per field is shown at right. Data are representative of 5 independent fields of view per group (*n* = 5). Scale bars: 90 μm (overview) and 20 μm (insets). (C) Effects of BspA-deficiency on spleen morphology and weight following *Brucella* infection. 6-8-weeks-old BALB/c mice were intraperitoneally injected with *BspA-Flag*, Δ*BspA* or CΔ*BspA* strains (3 × 10^5^ CFU). Spleens were harvested 21 days post-infection for gross morphology and weight measurements. (D) Bacterial burden in spleens. Spleen tissues from mice infected with *BspA-Flag*, Δ*BspA* or CΔ*BspA* strains (3 × 10^5^ CFU) were harvested 21 days post-infection for bacterial load quantification. (E) Effects of BspA-deficiency on *Brucella*-induced transcription of inflammatory genes in spleen. Spleen tissues from mice infected with *BspA-Flag*, Δ*BspA* or CΔ*BspA* strains (3 × 10^5^ CFU) were harvested 21 days post-infection and subjected to RT-qPCR analysis of mRNA levels of the indicated genes. (F) Histopathological analysis of spleens. Spleen tissues from mice infected with *BspA-Flag*, Δ*BspA* or CΔ*BspA* strains (3 × 10^5^ CFU) were harvested 21 days post-infection. Tissue sections were stained with hematoxylin and eosin (H&E) for histological examination. Quantification of multinucleated giant cells (MGCs) per field is shown at right. Data are representative of 5 independent fields of view per group (*n* = 5). Scale bars: 1.25 mm (overview) and 0.1 mm (insets). Data shown in (C-E) are mean ± SD, Mock, *n* = 3; *BspA-Flag*, Δ*BspA* or CΔ*BspA*, *n* = 6 independent samples. These experiments were repeated for at least twice with similar results. ns, nonsignificant, ∗*P* < 0.05, ∗∗*P* < 0.01.

We next investigated the roles of BspA in *Brucella*-induced pathogenesis. At 21 days post-infection, gross examination indicated that spleens from *BspA-Flag*- and CΔ*BspA*-infected mice were markedly larger in size and higher in weights than those from Δ*BspA*-infected mice (Fig. 7(C)), suggesting that BspA is responsible for *Brucella*-induced splenomegaly. Bacterial loads in the spleens of mice infected with the Δ*BspA* strain were significantly reduced compared to that of *BspA-Flag*- and *CΔBspA*-infected mice (Fig. 7(D)). RT-qPCR analysis and hematoxylin and eosin (H&E) staining of spleens showed that deficiency of BspA led to lower mRNA levels of inflammatory cytokines (IL-1β, TNF, CXCL10) (Fig. 7(E)) and reduced formation of multinucleated giant cells (MGCs) (Fig. 7(F)), which serve as hallmarks of *Brucella*-induced inflammatory response (Brooks et al., 2019). Collectively, these results suggest that deficiency of BspA reduces *Brucella* loads, *Brucella*-induced parthanatos and inflammatory response in mice, highlighting a critical role of BspA in pathogenicity of *Brucella*.

### 2.8 Olaparib is a potential therapeutic drug for brucellosis

Our earlier experiments indicated that olaparib effectively inhibited BspA-induced host cellular parthanatos, a key pathogenic mechanism of *Brucella*. Therefore, we sought to investigate the potential of olaparib as a therapeutic intervention for brucellosis. The results indicated that olaparib treatment attenuated *Brucella*-induced splenomegaly and reduced bacterial loads in the spleens of infected mice (Fig. 8(A) and (B)). H&E staining and RT-qPCR analysis indicated that olaparib suppressed *Brucella*-induced formation of MGCs (Fig. 8(C)) and transcription of inflammatory cytokines (IL-1β, TNF, and CXCL10) (Fig. 8(D)) in the spleens of infected mice. These results suggest that olaparib effectively attenuates *Brucella* virulence and *Brucella*-induced inflammatory response, highlighting its potential as a therapeutic agent for brucellosis.

**Fig. 8:**
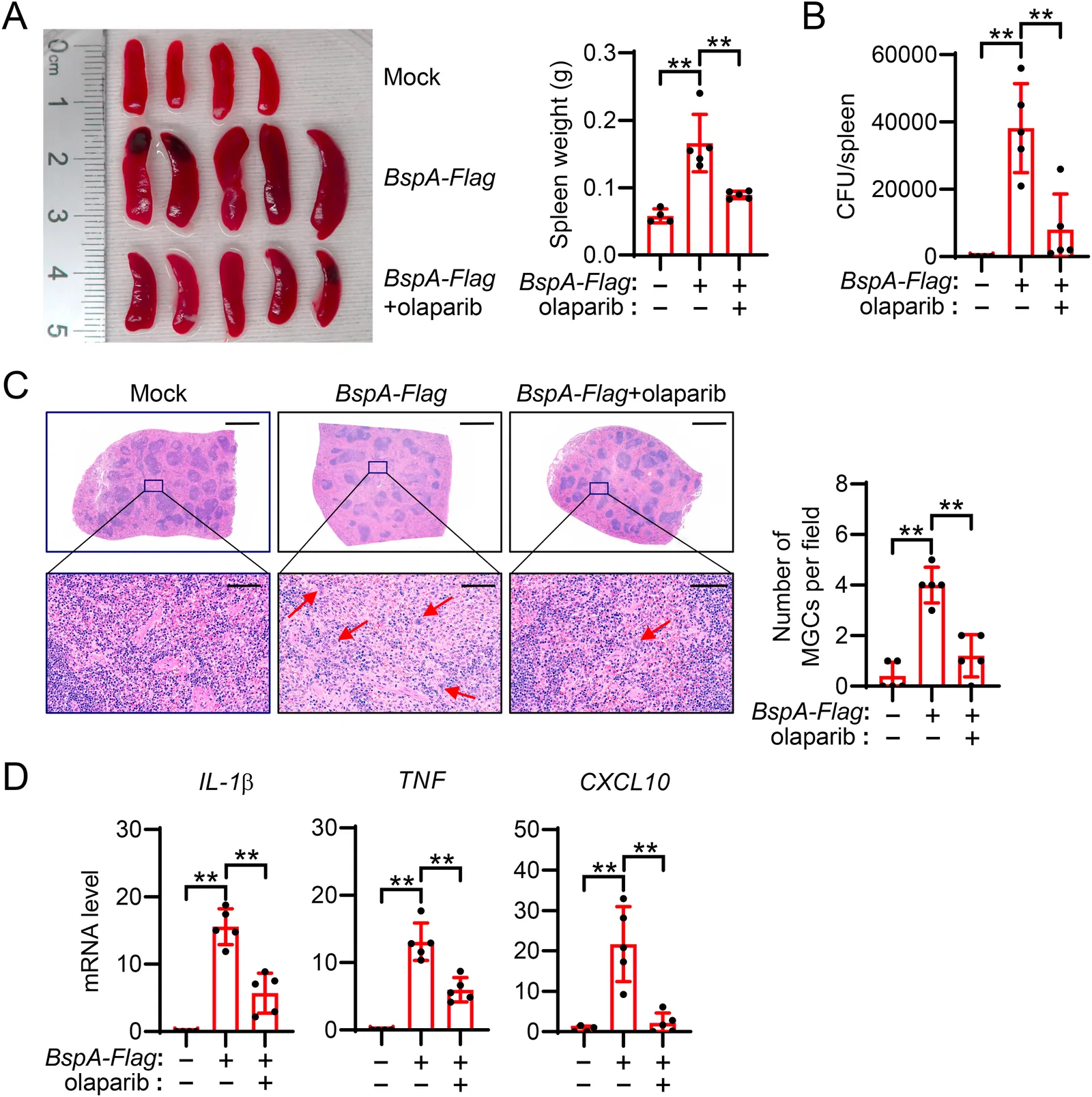
**Olaparib inhibits the pathogenicity of*****Brucella*****.** (A) Effects of olaparib on spleen morphology and weight following *Brucella* infection. 6-8-weeks-old BALB/c mice were intraperitoneally injected with *BspA-Flag* strain (3 × 10^5^ CFU) in the absence or presence of olaparib (25 mg/kg). Spleens were harvested 7 days post-infection for gross morphology and weight measurements. (B) Bacterial burden in spleens. Spleen tissues from mice infected with *BspA-Flag* strain (3 × 10^5^ CFU) in the absence or presence of olaparib (25 mg/kg) were harvested 7 days post-infection for bacterial load quantification. (C) Histopathological analysis of spleens. Spleen tissues from mice infected with *BspA-Flag* strain (3 × 10^5^ CFU) in the absence or presence of olaparib (25 mg/kg) were harvested 7 days post-infection. Tissue sections were stained with H&E for histological examination. Quantification of MGCs per field is shown at right. Data are representative of 5 independent fields of view per group (*n* = 5). Scale bars: 1.25 mm (overview) and 0.1 mm (insets). (D) Effects of olaparib on *Brucella*-induced transcription of inflammatory genes in spleen. Spleen tissues from mice infected with *BspA-Flag* strain (3 × 10^5^ CFU) in the absence or presence of olaparib (25 mg/kg) were harvested 7 days post-infection and subjected to RT-qPCR analysis of mRNA levels of the indicated genes. Data shown in (A), (B) and (D) are mean ± SD, Mock, *n* = 4; *BspA-Flag* or *BspA-Flag* + olaparib, *n* = 5 independent samples. Data shown in (C) are mean ± SD (*n* = 5). These experiments were repeated for at least twice with similar results. ∗∗*P* < 0.01.

## 3 Discussion

Intracellular pathogens usually exploit host cell death to facilitate their escape and dissemination, while also inducing tissue damage that exacerbates pathogenicity (Qiang et al., 2023; Sun et al., 2015; Zhao et al., 2025). Although previous studies have reported that *Brucella* infection can trigger macrophage death, the precise molecular mechanisms and the role of this process in *Brucella* pathogenesis remain poorly understood (Barquero-Calvo et al., 2015; Chen et al., 2011). Therefore, investigating the key pathways and molecular mechanisms underlying *Brucella*-induced host cell death, as well as clarifying its physiological relevance during infection, is essential for advancing our understanding of *Brucella* pathogenicity and for developing novel therapeutic strategies against brucellosis.

Our findings demonstrate that *Brucella*-induced host cell death is markedly alleviated by the Cu chelator TTM and the PARP-1 inhibitor olaparib, which are commonly used to block cuproptosis and parthanatos respectively. Further results reveal that *Brucella* infection does not trigger mitochondrial Cu overload, but induces hallmark events of parthanatos, including accumulation of PAR and formation of γ-H2AX. To further define the predominant mode of cell death induced by *Brucella* infection, we generated PARP-1- or FDX1-deficient cell lines to selectively block parthanatos or cuproptosis. The results demonstrate that deficiency of PARP-1, but not FDX1, effectively suppresses *Brucella*-induced cell death. Collectively, these findings suggest that *Brucella* primarily induces parthanatos rather than cuproptosis.

Through a systematic screen of known *Brucella* effector proteins, we identified the secreted protein BspA as a potent inducer of host cell death. Cell death induced by BspA is significantly inhibited by TTM and olaparib. Deficiency of PARP-1, but not FDX1, effectively suppresses BspA-induced cell death. Moreover, ectopic expression of BspA triggers hallmark events of parthanatos. Deficiency of BspA significantly attenuates *Brucella*-induced cell death and hallmark events of parthanatos. These results suggest that BspA is a key effector protein mediating *Brucella*-induced parthanatos of host cells. To further evaluate whether BspA-induced parthanatos is dependent on intracellular free Cu, we established a cellular model with low intracellular free Cu levels by overexpressing the Cu efflux transporter ATP7B. Under these conditions, BspA-induced cell death is markedly inhibited compared to the control group. These results demonstrate that *Brucella* induces parthanatos of host cells through the secreted protein BspA in a manner dependent on intracellular free Cu, which explains why *Brucella*- or BspA-induced parthanatos is effectively blocked by TTM.

Our findings demonstrate that BspA possesses the ability to bind Cu. Analysis of prokaryotically purified BspA reveals that BspA predominantly exists as soluble aggregates larger than 400 kDa, organized into membranous structures with an approximate diameter of 30 nm. Upon binding to Cu, these membranous structures are disrupted, leading to the formation of insoluble large irregular aggregates. In host cells, BspA is localized to the ER and mitochondria, where its binding to free Cu, leading to the formation of large insoluble irregular aggregates and disruption of ER and mitochondrial membrane structures accompanied by pronounced organelle swelling. The disruption of mitochondrial membrane integrity by BspA (upon Cu binding) leads to excessive production of ROS, a key trigger of parthanatos in host cells. In addition, *Brucella* infection induces ER–mitochondria membrane contacts in a manner dependent on the interaction between ER-located BspA and mitochondrial PTPIP51. Further investigation reveals that the disruption of ER structures by BspA leads to extensive Ca^2+^ leakage, which is subsequently taken up by mitochondria in a manner dependent on the ER–mitochondria contacts. Depletion of PTPIP51 inhibits approximately three-quarters of the mitochondrial Ca^2+^ elevation, while only reduces about one-third of cell death and ROS production. These results suggest that mitochondrial Ca^2+^ overload may induce only part of the mitochondrial ROS, and ROS arising from BspA-induced mitochondrial damage also contributes to the execution of parthanatos. Therefore, our studies suggest that BspA triggers parthanatos via two routes: 1) mitochondrial located BspA disrupts mitochondrial structures upon Cu binding, leading to ROS production and parthanatos; 2) the ER-located BspA interacts with the mitochondrial PTPIP51, which facilitates ER–mitochondria contacts and Ca^2+^ transport from the ER to mitochondria upon BspA-mediated disruption of these membrane structures, leading to increased ROS production and parthanatos.

Our data further support a critical role of BspA-induced parthanatos in pathogenicity of *Brucella in vivo*. Deficiency of BspA markedly inhibits splenomegaly, reduces bacterial burden, and suppresses the formation of multinucleated giant cells and the transcription of proinflammatory genes in the spleen of *Brucella*-infected mice. These findings suggest that BspA-induced parthanatos is not merely a byproduct of infection but rather a key contributor to *Brucella*-induced tissue pathology, immune modulation and dissemination of *Brucella in vivo*. The simplest explanation for our observations is that BspA-induced parthanatos is important for replicated *Brucella* to disseminate and infect neighboring and distant cells, therefore amplifying its infection and inflammatory response, leading to *Brucella* virulence. Importantly, the observation that olaparib, a clinically approved PARP-1 inhibitor used as a frontline cancer therapy (Murai et al., 2012; Zhou et al., 2025), effectively reduced splenic inflammation and bacterial burden, underscores the translational potential of olaparib and targeting parthanatos for the treatment of brucellosis.

## 4 Materials and methods

### 4.1 Reagents, antibodies, cells and *Brucella* strains

Trypsin (C100C1, NCM Biotech), puromycin (AMR-J593, VWR), Lipofectamine RNAiMAX (13778150, Invitrogen), mitochondria isolation kit (89874, Thermo Scientific), genomic DNA extraction kit (DP304-02, TIANGEN), protein A + G agarose (P2055, Beyotime), PMSF (P7626, Sigma), SYBR Green Supermix (Q312, Vazyme), HiScript II Select RT SuperMix for qPCR (R323, Vazyme), CuCl_2_ (451665, Sigma), ferrostatin-1 (T6500, TargetMol), necrostatin-1 (T1847, TargetMol), Q-VD-OPH (T0282, TargetMol), and tetrathiomolybdate, olaparib, staurosporine, aprotinin, leupeptin, β-glycerophosphate disodium salt and sodium orthovanadate (HY-128530, HY-10162, HY-15141, HY-P0017, HY-18234A, HY-126304 and HY-D0852 respectively; MCE) were purchased from the specified manufacturers.

Mouse monoclonal antibody against HA (66006, Proteintech), rabbit monoclonal antibody against Myc (AE070, ABclonal), mouse monoclonal antibody against Flag (AP0530, Sigma), mouse and rabbit IgG (I5381 and I5006, Sigma), poly/mono-ADP ribose (D9P7Z) rabbit mAb (89190, Cell Signaling Technology), DLAT (4A4-B6-C10) mouse mAb (12362, Cell Signaling Technology), TOMM20 mouse mAb (66777-1-Ig, Proteintech), β-Tubulin mouse mAb (GB15140-100, Servicebio), Lamin B1 Rabbit pAb (A1910, ABclonal), PARP-1 rabbit mAb (A27791, ABclonal), γ-H2AX rabbit mAb (AP1555, ABclonal), H2AX rabbit mAb (A2082, ABclonal), FDX1 rabbit mAb (A20895, ABclonal), PTPIP51 rabbit mAb (A5820, ABclonal), Caspase3 rabbit mAb (A19654, ABclonal), AIF rabbit mAb (A2568, ABclonal) and Cleaved Caspase-3 (Asp175) (5A1E) rabbit mAb (9664, Cell Signaling Technology) were used in this study. Fluorescent secondary antibodies included Alexa Fluor 594-conjugated anti-mouse IgG (H + L), F(ab')_2_ fragment (8890, Cell Signaling Technology), Alexa FluorTM 488-conjugated goat anti-rabbit IgG (H + L) (A11008, Invitrogen) and Alexa FluorTM 405-conjugated goat anti-rabbit IgG (H + L) (A31556, Invitrogen). The anti-Omp28 antibody was previously described (Zhi et al., 2024).

HEK293T, HeLa and RAW264.7 cells were obtained from the American Type Culture Collection (ATCC). Cells were maintained in Dulbecco's Modified Eagle Medium (DMEM; C11965500BT, Gibco) supplemented with 10% fetal bovine serum (FBS; SA211.02, Cellmax) and 1% penicillin-streptomycin (PB180120, Pricella) at 37 °C in a humidified incubator with 5% CO_2_.

*B. abortus* S2308 was previously described (Zhi et al., 2024). Mutant *B. abortus* S2308 were engineered based on this parental strain.

### 4.2 Plasmids

Expression plasmids encoding *Brucella*-secreted proteins, Flag-tagged GFP, HA-tagged ATP7B, and Myc-tagged VDAC1, MFN2, PTPIP51 and TOMM40 were constructed using standard molecular cloning techniques.

### 4.3 Gene knockout by the CRISPR-cas9 methods

Double-stranded oligonucleotides targeting the indicated gene sequences were designed using the online CRISPR design tool CHOPCHOP and individually inserted into the lentiCRISPR v2 vector (52961, Addgene). The recombinant plasmids (20 μg) were individually co-transfected with the packaging plasmids psPAX2 (12 μg) and pCMV-VSV-G (10 μg) into HEK293T cells in a 10 cm dish. At 48 h after transfection, lentiviral supernatants were harvested and used to infect RAW264.7 or HEK293T cells (3 × 10^5^). The infected cells were selected with puromycin (4 μg/mL for RAW264.7 cells and 2 μg/mL for HEK293T cells) for 6 days to establish indicated genes-deficient cell lines. The following sgRNA sequences were used:

Human g*PARP-1*: 5′-GTCCACCATCTTCTTGGAC-3′;

Human g*FDX1*: 5′-CAGCGGCCTGCTGAGGAAC-3′;

Mouse g*Parp-1*: 5′-CGACGCTTATTACTGTACTG-3′;

Mouse g*Fdx1*: 5′-ACCTTCCGACAGGGTTATGG-3′.

### 4.4 RNAi experiments

Small interfering RNAs (siRNAs) targeting the indicated genes were synthesized and transfected into HEK293T or HeLa cells using Lipofectamine RNAiMAX reagent according to the manufacturer's instructions. The following siRNA sequences were used:

Human *BAP31*: 5′-CAGCACUAAGCAAAAACUAGA-3′;

Human *IP3R3*: 5′-GCAAGAACCAGGAGCACAU-3′;

Human *MFN2*: 5′-CCCUCAACUAUGACCUAAA-3′;

Human *VAPB*: 5′-GCUCUUGGCUCUGGUGGUU-3′;

Human *PTPIP51*: 5′-CCUUAGACCUUGCUGAGAU-3′.

### 4.5 RT-qPCR

Total RNA was extracted from cells using RNAiso Plus reagent (9109, Takara) according to the manufacturer's protocol. The cDNA was synthesized by reverse transcription using HiScript II Select RT SuperMix (R323, Vazyme). qPCR was performed using SYBR Green Supermix on a Bio-Rad Real-Time PCR System with gene-specific primers. Relative mRNA expression levels of the indicated genes were calculated using the 2^−ΔΔCt^ method and normalized to the expression of human *GAPDH or* mouse *Gapdh* as internal controls.

The sequences of qPCR primers were as follows:

Human *GAPDH*: 5′-GACAAGCTTCCCGTTCTCAG-3′,

5′-GAGTCAACGGATTTGGTCGT-3′;

Human BAP31: 5′-AGAACAGGAGCCTGAAGGCTGA-3′,

5′-AAGCGGTCGTACTCCTTGGTGA-3′;

Human *IP3R3*: 5′-CCACAGGCAACGAGGGTTC-3′,

5′-CCCGGCATTGACAGGATTCA-3′;

Human *MFN2*: 5′-ATTGCAGAGGCGGTTCGACTCA-3′,

5′-TTCAGTCGGTCTTGCCGCTCTT-3′;

Human *VAPB*: 5′-AGGTTATGGAAGAATGTAAGAGGC-3′,

5′-GTTGCTCTGCACTGTCTTCCTC-3′;

Human *PTPIP51*: 5′-TCACTGAGGAGGTGAGCGAGAA-3′,

5′-CAGGTGACAGTCAGCACTCTCA-3′;

Mouse *Gapdh*: 5′-ACGGCCGCATCTTCTTGTGCA-3′,

5′-ACGGCCAAATCCGTTCACACC-3′;

Mouse *IL-1β*: 5′-AAAGCCTCGTGCTGTCGGACC-3′,

5′-CAGGGTGGGTGTGCCGTCTT-3′;

Mouse *TNF*: 5′-GGTGATCGGTCCCCAAAGGGATGA-3′,

5′-TGGTTTGCTACGACGTGGGCT-3′;

Mouse *CXCL10*: 5′-ATCATCCCTGCGAGCCTATCCT-3′,

5′-GACCTTTTTTGGCTAAACGCTTTC-3′.

### 4.6 Generation of *BspA-Flag*, Δ*BspA* and CΔ*BspA* strains

Mutant *B. abortus* S2308 were constructed by double homologous recombination. The *BspA-Flag* strain was constructed by replacing the endogenous *BspA* gene with an epitope-tagged version. Briefly, ∼400 base pairs homologous sequences flanking both sides of the target gene were amplified and cloned into the kanamycin-resistant vector pUC19 containing the *sacB* gene as a counter-selectable marker (pUC19-*sacB*-LA-*Flag*-RA). The constructs were verified by restriction enzyme digestion and Sanger sequencing. Recombinant plasmids were electroporated into competent *B. abortus* S2308 cells. Following 24 h of recovery in antibiotic-free tryptic soy broth (TSB), transformants were selected on tryptic soy agar (TSA) plates containing kanamycin to identify colonies harboring single-crossover (first homologous recombination) events. For selection of double-crossover (second recombination) mutants, colonies were plated on TSA supplemented with 15% sucrose. Due to the presence of the *sacB* gene, which encodes the sucrose-sensitive enzyme levansucrase, only plasmid-excised recombinants could survive under these conditions.

The Δ*BspA* strain was generated by deleting the *BspA-Flag* gene via a similar strategy. A recombinant plasmid (pUC19-*sacB*-LR-RA) was constructed and introduced into *B. abortus* S2308 cells, followed by the same selection procedures described above.

For CΔ*BspA* strain, the *BspA* gene was cloned into the shuttle vector pBBR1MCS-2 to generate pBBR1MCS-2-*BspA*-*Flag*. The plasmid was electroporated into competent cells of the Δ*BspA* strain. After 24 h of recovery in antibiotic-free TSB, complemented strains were selected on TSA containing kanamycin.

The generation of *BspA-Flag*, Δ*BspA* and CΔ*BspA* strains were confirmed by immunoblotting analysis.

### 4.7 Confocal microscopy

HeLa cells were seeded on coverslips in 24-well plates and transfected with the indicated plasmids for 24 h or 48 h. The Golgi apparatus, endoplasmic reticulum (ER), and mitochondria were labeled with GM130-RFP, CANX-GFP, and COX8A-RFP, respectively. The cells were fixed with 4% paraformaldehyde for 30 min and then permeabilized with 0.1% Triton X-100 for 15 min. Subsequently, the cells were blocked in 1% bovine serum albumin for 1 h and then stained with the indicated primary and secondary antibodies for 2 h. Imaging of cells was carried out using a Zeiss confocal laser scanning microscope 980 under standard settings.

### 4.8 Co-immunoprecipitation

The cells were lysed in lysis buffer (20 mM Tris-HCl pH 7.4, 1% NP-40, 150 mM NaCl, 1 mM EDTA and protease inhibitors) at 4 °C for 10 min, followed by sonication. The lysates were centrifuged at 13 000 rpm for 10 min at 4 °C, and the resulting supernatants were incubated with the indicated antibodies for immunoprecipitation. The antibody-protein complexes were captured with protein A/G beads and then washed three times with high-salt washing buffer (750 mM NaCl, 50 mM Tris-HCl, pH 7.4). The bound proteins were separated by SDS-PAGE and analyzed by immunoblotting with the indicated antibodies.

### 4.9 Analysis of LDH release

RAW264.7 cells were seeded in 24-well plates and allowed to attach overnight. The cells were infected with *Brucella* at the indicated MOI and time points. At each time point, 100 μL of cell-free supernatant from each well was gently centrifuged at 3000 rpm for 5 min and transferred to a new 96-well plate for LDH measurement. For the maximum LDH release control, matched wells were lysed with 1% Triton X-100 for 10 min at room temperature prior to centrifugation and supernatant collection. LDH activity was measured using a commercial LDH cytotoxicity kit (C0016, Beyotime) according to the manufacturer's protocol, and absorbance was read at 490 nm using a microplate reader. All conditions were assayed in triplicate. Percent cytotoxicity was calculated as: % LDH release = (OD_sample_−OD_blank_)/(OD_maximum_−OD_blank_)×100%.

### 4.10 CCK**-**8 assay

HEK293T cells were seeded in 24-well plates and allowed to attach overnight. The cells were transfected with the indicated plasmids for the specified times. Subsequently, 50 μL of CCK-8 reagent (C0005, TargetMol) was added directly to each well. Plates were incubated at 37 °C for 2 h. Absorbance was read at 450 nm using a microplate reader. All conditions were set up in triplicate wells and experiments were repeated at least three times. Cell viability (%) was calculated as: Fold Viability = (OD_sample_ − OD_blank_)/(OD_control_ − OD_blank_) × 100%.

### 4.11 Measurement of ROS levels

The cellular and mitochondrial ROS levels were assessed by fluorescence microscopy using commercial detection kits. HEK293T cells were seeded in 24-well plates at a density of 2 × 10^5^ cells per well and transfected with the BspA plasmids for 24 h. The cells were loaded with the fluorescent ROS indicator DCFH-DA (S0033, Beyotime; 10 μM) or MitoSOX Red (HY-D1055, MCE; 3 μM) at 37 °C for 30 min in the dark to measure cellular and mitochondrial ROS levels, respectively. After being washed twice with PBS, the cells were directly observed by fluorescence microscopy. Mean fluorescence intensity was quantified from at least five randomly selected fields using ImageJ software and normalized to the control group.

### 4.12 Measurement of NO levels

The intracellular NO level was quantified by microplate reader using a commercial detection kit (S0021, Beyotime), following the manufacturer's protocol. HEK293T cells were seeded in 24-well plates at a density of 2 × 10^5^ cells per well and transfected with the BspA plasmids for 24 h. The cells were lysed in NP-40 buffer, and the lysates were centrifuged at 12 000×*g* for 10 min to collect the supernatants. NO concentration was then determined using the Griess reaction. Absorbance was measured at 540 nm using a microplate reader, and NO concentration was calculated based on a standard curve generated with sodium nitrite. Relative NO concentrations in the indicated groups were normalized to those in the control group.

### 4.13 Measurement of mitochondrial membrane potential

The mitochondrial membrane potential (Δψm) of host cells was assessed by flow cytometry using commercial detection kits (HY-D1075, MCE). HEK293T cells were seeded in 24-well plates at a density of 2 × 10^5^ cells per well and transfected with the BspA plasmids for 24 h. The cells were incubated with 50 nM indicator DiIC1(5) for 30 min at 37 °C in the dark. After staining, cells were washed twice with PBS, and immediately analyzed by flow cytometry. The mean fluorescence intensity of DiIC1(5) was quantified to assess changes in Δψm and normalized to the values of the control group.

### 4.14 Measurement of ATP levels

The intracellular ATP level was studied by microplate reader using a commercial detection kit (S0026, Beyotime), following the manufacturer's protocol. Briefly, RAW264.7 cells were seeded in 24-well plates at a density of 2 × 10^6^ cells per well and infected with *Brucella* at the indicated MOI and time points. After infection, cells (1 × 10^6^) were lysed in the provided lysis buffer, and the clarified lysates were mixed with the luciferase reaction reagent. Luminescence intensity was immediately measured using a luminometer, and ATP concentrations were calculated based on an ATP standard curve and then normalized to the values of the control group.

### 4.15 Measurement of intracellular and mitochondrial Ca^2+^ levels

The intracellular and mitochondrial Ca^2+^ levels were measured by fluorescence microscopy using commercial detection kits. HEK293T cells were seeded in 24-well plates at a density of 2 × 10^5^ cells per well and transfected with the BspA plasmids for 24 h. The cells were incubated with 5 μM Fluo-4 AM (S1061, Beyotime) or 5 μM Rhod-2 AM (S1062, Beyotime) for 30 min at 37 °C in the dark to measure intracellular or mitochondrial Ca^2+^ levels, respectively. After being washed twice with PBS, the cells were directly observed by fluorescence microscopy. The fluorescence intensity of Fluo-4 or Rhod-2 was quantified using ImageJ software to assess relative Ca^2+^ levels and then normalized to the values of the control group.

### 4.16 Copper affinity binding assay

Profinity M IMAC resin (1560121, Biorad) was washed and loaded with 0.3 M of CuCl_2_ or H_2_O based on the manufacturer's protocol. HEK293T cells ectopically expressing BspA were lysed in RIPA buffer (50 mM Tris-HCl pH 7.4, 150 mM NaCl, 1% Triton X-100, 1% sodium deoxycholate, and 0.1% SDS). The clarified lysates were applied to the Cu^2+^-loaded or Cu^2+^-free resins and incubated for 1 h at 4 °C with gentle rotation. The columns were washed three times with PBS to remove unbound proteins and then eluted with 300 mM imidazole (elution buffer). Eluted proteins were analyzed by immunoblotting with the indicated antibodies.

### 4.17 Measurement of mitochondrial Cu levels by ICP-MS

RAW264.7 cells were seeded in 6-well plates at a density of 4 × 10^6^ cells per well and infected with *Brucella* at an MOI of 200 for 48 h. Following cell harvesting, mitochondria were isolated using a mitochondrial isolation kit (89874, Thermo Scientific) based on the manufacturer's protocol and subsequently digested in nitric acid to quantify mitochondrial Cu levels by ICP-MS (Wuhan ProNets Testing Technology Co., Ltd).

### 4.18 Transmission electron microscopy

HeLa cells were seeded in 10 cm dishes, then infected with *Brucella* at an MOI of 200. At 48 h post-infection, the cells were washed twice with PBS and then fixed with 2.5% glutaraldehyde at 4 °C for 30 min. Fixed cell pellets were dehydrated in a series of acetone solutions and embedded in epoxy resin. Ultrathin sections were prepared, stained with uranyl acetate for 1 min, and examined by transmission electron microscopy using a Hitachi HT7700 microscope.

### 4.19 BspA expression and purification

BspA with an N-terminal Flag tag was cloned into a pET22b vector. This vector was transformed into SHuffle T7 competent *E. coli* cells (C3026J, New England Biolabs), and the cells were grown overnight in LB broth supplemented with 100 μg/mL ampicillin, shaking at 220 rpm and 37 °C. The next day, the saturated overnight culture was used to inoculate Terrific Broth supplemented with 100 μg/mL ampicillin, and cells were grown with shaking at 220 rpm at 37 °C. When cells reached an optical density (OD600) of 0.6, the expression of BspA was induced by the addition of 1 mM IPTG, and the incubation temperature was reduced to 25 °C for 24 h. Cells were harvested by centrifugation for subsequent purification of BspA. The induced bacterial cells were thawed on ice and resuspended in the lysis buffer containing 50 mM Tris (pH 8.0), 150 mM NaCl, and 1×protease inhibitor cocktail (HY-K0010, MCE), and then lysed by sonication. The soluble supernatant was separated by centrifugation at 18 000×*g* for 30 min and incubated with Anti-DYKDDDDK G1 Affinity Resin (L00432, Genscript) for 2 h. After extensive washing, BspA was eluted with 20 mM Tris (pH 8.0), 100 mM NaCl, 100 μg/mL Flag peptide, and 1×protease inhibitor cocktail.

### 4.20 Size-exclusion chromatography

Size-exclusion chromatography was performed using an FPLC system (Cytiva) equipped with a Superdex 200 Increase 10/300 GL column. The column was equilibrated with buffer containing 20 mM Tris-HCl (pH 8.0) and 100 mM NaCl at 4 °C and operated at a flow rate of 0.5 mL/min. The purified BspA protein, either incubated or not incubated with 10 μM Cu^2+^, was centrifuged at 12 000×*g* for 10 min at 4 °C and filtered through a 0.22 μm membrane prior to loading. Protein elution was monitored by UV absorbance at 280 nm and collected using an AKTA pure chromatography system (Cytiva).

### 4.21 Nucleocytoplasmic separation and mitochondria isolation assays

Nuclear extracts were prepared using Nuclear and Cytoplasmic Protein Extraction Kit (P0028, Beyotime) according to the manufacturer's instructions. Mitochondrial proteins were isolated and extracted using a cell mitochondria isolation kit (89874, Thermo Scientific) according to the manufacturer's instructions.

### 4.22 *Brucella* infection *in vitro*

RAW264.7 or HeLa cells were seeded in 24-well plates at a density of 5 × 10^5^ cells per well and infected with *Brucella* at an MOI of 200 for 4 h. Following infection, the cells were incubated with medium containing 50 μg/mL gentamicin for 1 h to eliminate extracellular bacteria, and subsequently maintained in medium with 25 μg/mL gentamicin to prevent reinfection. For analysis of protein expression, infected cells were harvested at the indicated time points post-infection, and lysed in SDS sample buffer for immunoblotting analysis. To quantify intracellular bacterial loads, cells were lysed at indicated time points using 0.5 mL PBS containing 0.5% Triton X-100. Lysates were serially diluted and plated on TSA to determine CFUs.

### 4.23 Animal experiments

Male BALB/c mice (6-8 weeks-old) were intraperitoneally infected with 3 × 10^5^ CFU of *Brucella*. At 21 days post-infection, bacterial burdens in spleens were determined by plating tissue homogenates on TSA plates. Spleens were also weighed to assess splenomegaly and processed for histological analysis including hematoxylin and eosin (H&E) staining and immunohistochemical staining with anti-poly/mono-ADP ribose (D9P7Z) rabbit mAb staining. For drug treatment, olaparib was dissolved in a solution containing 10% DMSO, 40% PEG300, 5% Tween-80, and 45% H_2_O, and administered intraperitoneally at a dose of 25 mg/kg one day before infection and then every other day thereafter.

### 4.24 Ethics statement

All animals were handled strictly with good animal practices according to the Animal Ethics Procedures and Guidelines of the People's Republic of China. The study was approved by the Animal Ethics Committee of the Lanzhou Veterinary Research Institute of the Chinese Academy of Agricultural Sciences (approval number: LVRIAEC-2025-015).

### 4.25 Data availability

All data reported in this paper are available in the paper or supplementary data.

### 4.26 Facility biosafety statement

All experiments with live *B. abortus* S2308 (CVCC788) or its derivatives were conducted at the Biosafety Level 3 (BSL-3) laboratory of the Lanzhou Veterinary Research Institute (LVRI), Chinese Academy of Agricultural Sciences (CAAS). This laboratory is accredited by the China National Accreditation Service for Conformity Assessment (CNAS) and Gansu Provincial Department of Agriculture and Rural Affairs.

### 4.27 Statistical analysis

Statistical analyses were performed using unpaired Student's *t*-test for two-group comparisons and one-way ANOVA followed by Tukey's multiple comparisons test for multiple-group comparisons. The number of asterisks represents the degree of significance with respect to P values. Statistical significance was set at *P* < 0.05, ∗ or *P* < 0.01, ∗∗.

## CRediT authorship contribution statement

**Li-Bo Cao:** Writing – review & editing, Writing – original draft, Validation, Supervision, Resources, Project administration, Methodology, Investigation, Funding acquisition, Formal analysis, Data curation, Conceptualization. **Jin-Ke Yang:** Methodology, Investigation, Data curation. **Xuehui Wang:** Methodology, Investigation, Data curation. **Xiao-Wen Wang:** Methodology, Investigation, Data curation. **Shanquan Wu:** Methodology, Investigation, Data curation. **Zhen-Zhen Li:** Methodology, Investigation, Data curation. **Jia-Jing Wang:** Methodology, Investigation, Data curation. **Dongsheng Lei:** Validation, Supervision, Resources, Project administration, Methodology, Investigation, Funding acquisition, Formal analysis, Data curation, Conceptualization. **Jian Xu:** Supervision, Resources, Project administration, Methodology, Funding acquisition, Formal analysis, Data curation, Conceptualization. **YueFeng Chu:** Supervision, Resources, Project administration, Methodology, Funding acquisition, Formal analysis, Data curation, Conceptualization. **Xin Wu:** Writing – review & editing, Writing – original draft, Validation, Supervision, Resources, Project administration, Methodology, Investigation, Funding acquisition, Formal analysis, Data curation, Conceptualization. **Yu-Lin Yang:** Writing – review & editing, Writing – original draft, Validation, Supervision, Resources, Project administration, Methodology, Investigation, Funding acquisition, Formal analysis, Data curation, Conceptualization.

## Declaration of competing interest

The authors declare that they have no known competing financial interests or personal relationships that could have appeared to influence the work reported in this paper.
