# Supplementary material for: Host cellular parthanatos induced by Brucella effector BspA is a critical determinant for its virulence
Source: Cell Insight. 2026 Sep 25;5(6):100376. doi: 10.1016/j.cellin.2026.100376 (PMC13734774; doi:10.1016/j.cellin.2026.100376)
Supplement: Supplementary file 1 [file mmc1.pdf]

Cao et al., Fig. S1

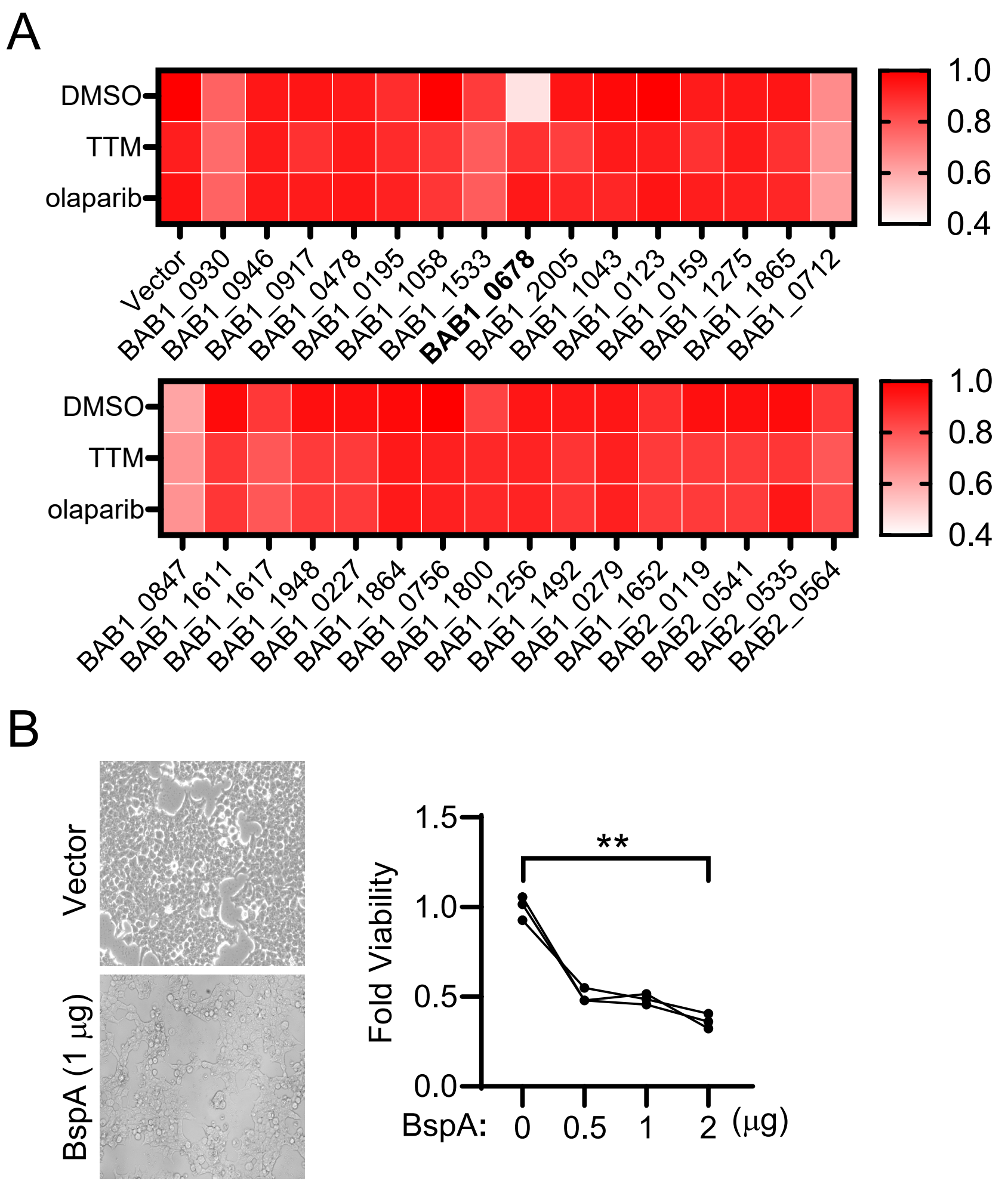

Fig. S1. Screening of *Brucella* effector proteins that induce parthanatos.

A. Screening of *Brucella*-secreted proteins that induce cell death, which can be independently inhibited by TTM and olaparib. HEK293T cells (5×10^5) were transfected with an empty vector or plasmids encoding *Brucella*-secreted proteins for 48 hours in the absence or presence of TTM (40 μM) or olaparib (20 μM), followed by CCK8 analysis.

B. Effects of BspA on cell viability. HEK293T cells (5×10^5) were transfected with BspA plasmids (0, 0.5, 1 and 2 μg) for 48 hours, followed by CCK8 analysis.

Data shown in A and B are mean ± SD (n = 3) from one representative experiment. All the experiments were repeated at least twice with similar results. **P<0.01.

Cao et al., Fig. S2

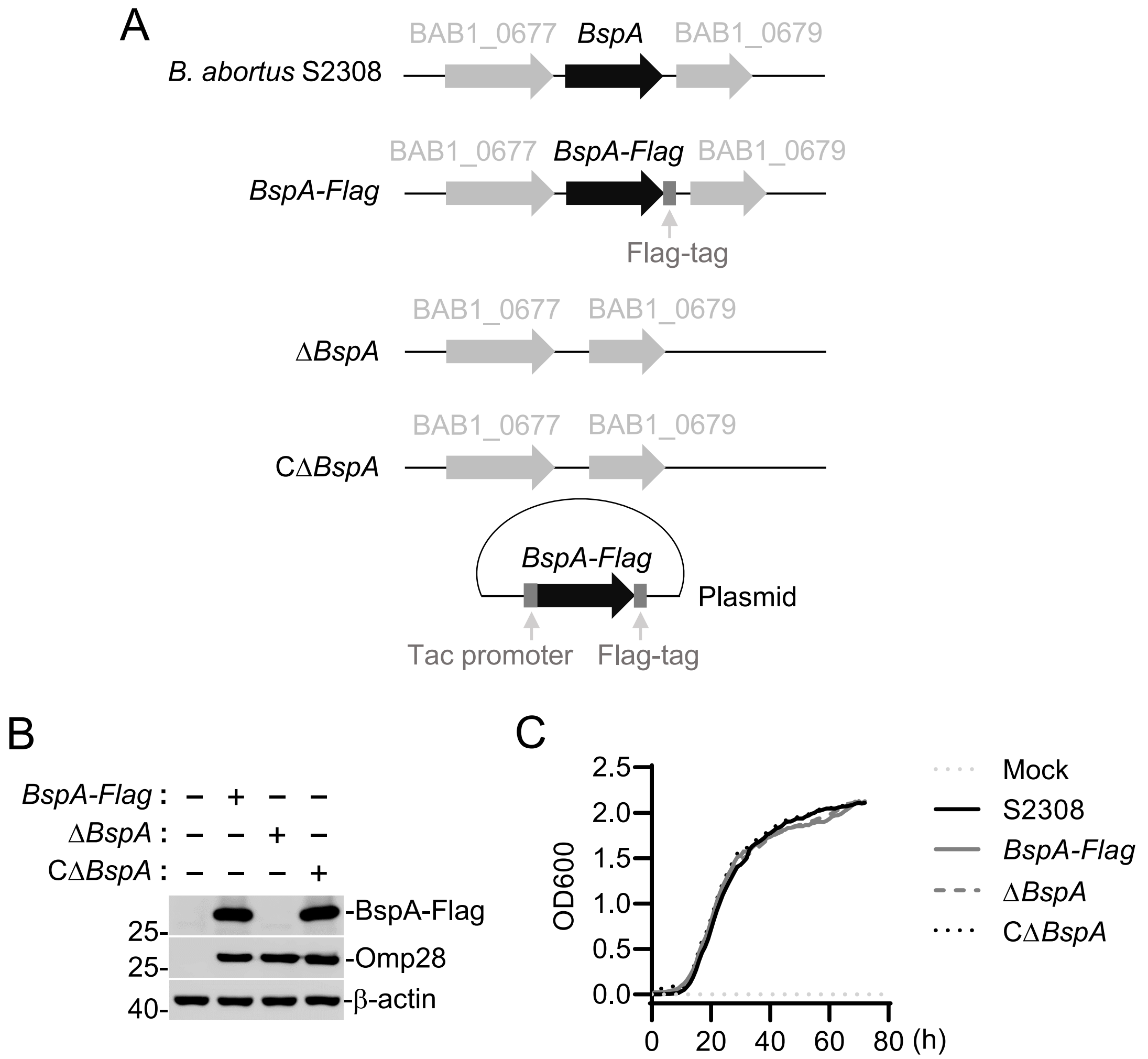

Fig. S2. Construction and characterization of *B. abortus* mutant strains.

A. Schematic diagrams of the generation of Flag-tagged *BspA* strain (*BspA-Flag*), *BspA*-deficiency strain (Δ*BspA*), and *BspA* complemented strain (CΔ*BspA*).

B. Immunoblotting verification of *BspA-Flag*, Δ*BspA* and CΔ*BspA* purity. RAW264.7 cells (2×10^5) were left uninfected or infected with *BspA-Flag*, Δ*BspA* or CΔ*BspA* strains (MOI = 200) for 48 hours, followed by immunoblotting analysis with the indicated antibodies.

C. Growth kinetics of wild-type, *BspA-Flag*, Δ*BspA* and CΔ*BspA B. abortus* strains in TSB medium. Bacteria were cultured to mid-log phase (OD600 = 0.6), diluted 1:100 in fresh TSB, and OD600 was measured every 1 hour over 72 hours.

All the experiments were repeated at least twice with similar results.

Cao et al., Fig. S3

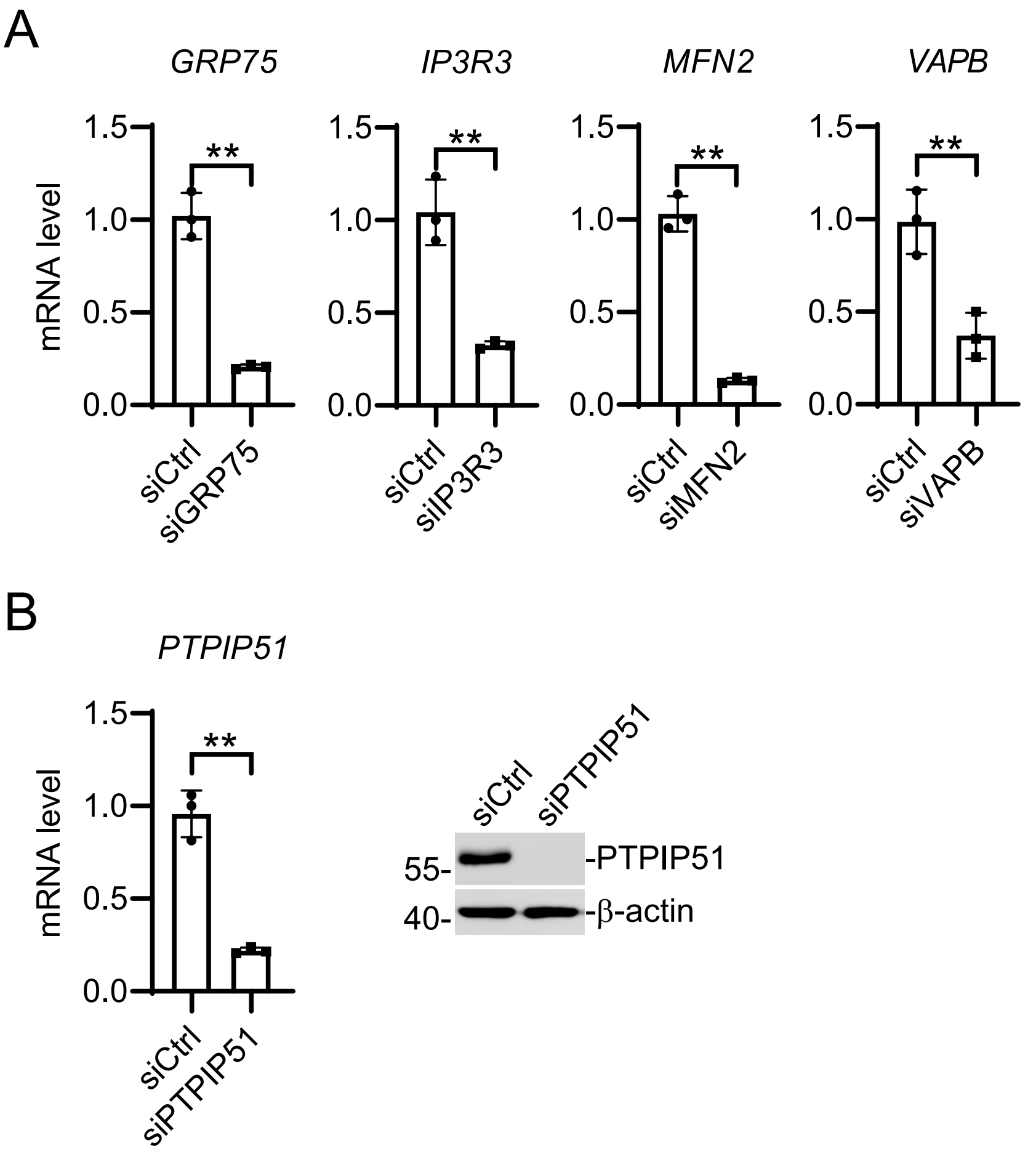

Fig. S3. Knockdown efficiency of proteins mediating ER-mitochondria contacts.

A. Detection of the knockdown efficiency of four classical ER-localized contact proteins. HEK293T cells (1×10^5) were transfected with the indicated siRNAs (50 nM) for 48 hours before RT-qPCR analysis of mRNA levels of the indicated genes.

B. Detection of the knockdown efficiency of PTPIP51. HEK293T cells (1×10^5) were transfected with the indicated siRNAs (50 nM) for 48 hours before RT-qPCR analysis of mRNA levels of the indicated genes (left) and immunoblotting analysis with the indicated antibodies (right)

Data shown in A and B are mean ± SD (n = 3) from one representative experiment. All the experiments were repeated at least twice with similar results. **P<0.01.

Cao et al., Fig. S4

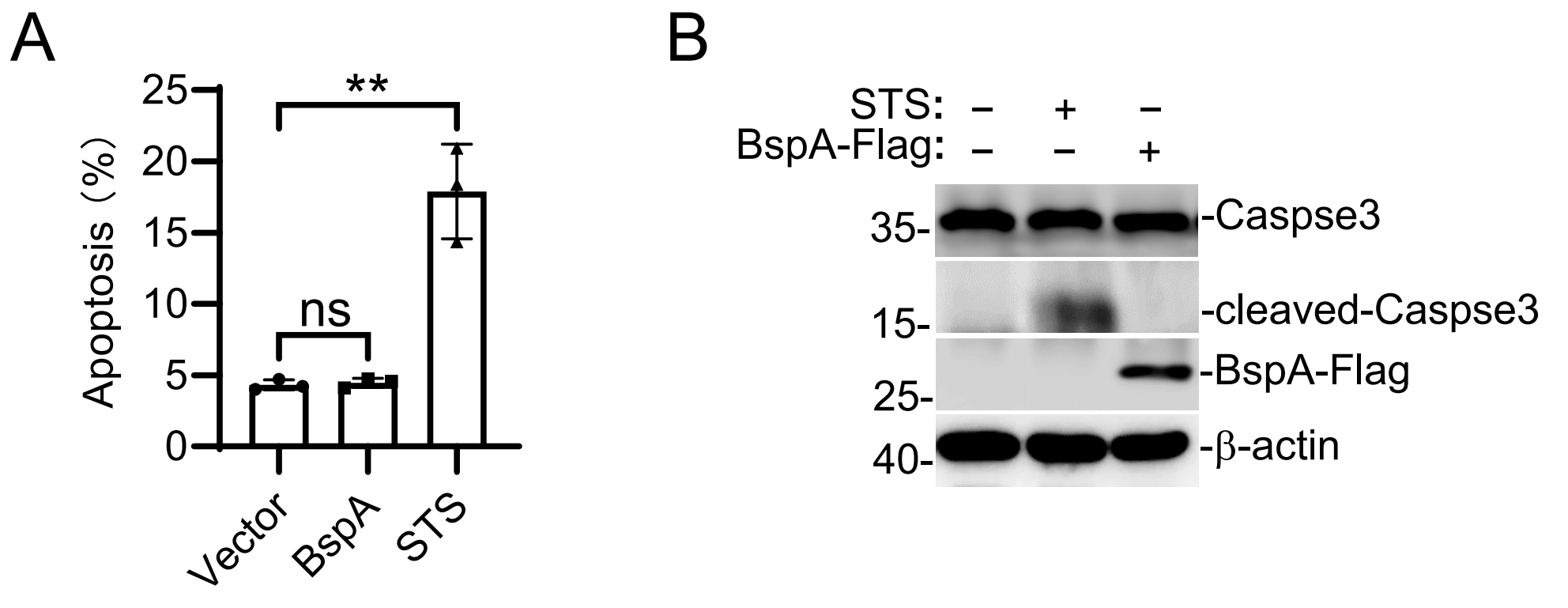

Fig. S4. BspA-induced mitochondrial Ca^{2+} overload does not trigger apoptosis. A-B. Effects of BspA on apoptosis of host cells. HEK293T cells (1×10^5) were transfected with the indicated plasmids (1 μg) for 36 hours or treated with staurosporine (STS, 5 μM) for 8 hours before apoptosis analysis by flow cytometry (A) or immunoblotting analysis with the indicated antibodies (B). Data shown in A is mean ± SD (n = 3) from one representative experiment. All the experiments were repeated at least twice with similar results. ns, nonsignificant,**P<0.01 .
